# Integrated single-cell transcriptome analysis of the tumor ecosystems underlying cervical cancer metastasis

**DOI:** 10.3389/fimmu.2022.966291

**Published:** 2022-12-09

**Authors:** Chunbo Li, Danyang Liu, Shimin Yang, Keqin Hua

**Affiliations:** ^1^ Department of Obstetrics and Gynecology, Obstetrics and Gynecology Hospital of Fudan University, Shanghai, China; ^2^ Department of Pathology, Obstetrics and Gynecology Hospital of Fudan University, Shanghai, China

**Keywords:** cervical cancer, single-cell sequencing, metastatic lymph node, tumor microenvironment, tumor heterogeneity

## Abstract

Cervical cancer (CC) is one of the most frequent female malignancies worldwide. However, the molecular mechanism of lymph node metastasis in CC remains unclear. In this study, we investigated the transcriptome profile of 51,507 single cells from primary tumors, positive lymph nodes (P-LN), and negative lymph nodes (N-LN) using single-cell sequencing. Validation experiments were performed using bulk transcriptomic datasets and immunohistochemical assays. Our results indicated that epithelial cells in metastatic LN were associated with cell- cycle-related signaling pathways, such as E2F targets, and mitotic spindle, and immune response-related signaling pathways, such as allograft rejection, IL2_STAT5_signaling, and inflammatory response. However, epithelial cells in primary tumors exhibited high enrichment of epithelial-mesenchymal translation (EMT), oxidative phosphorylation, and interferon alpha response. Our analysis then indicated that metastasis LN exhibited an early activated tumor microenvironment (TME) characterized by the decrease of naive T cells and an increase of cytotoxicity CD8 T cells, NK cells, FOXP3+ Treg cells compared with normal LN. By comparing the differently expressed gene of macrophages between tumor and metastatic LN, we discovered that C1QA+ MRC1^low^ macrophages were enriched in a tumor, whereas C1QA+ MRC1^high^ macrophages were enriched in metastatic LN. Finally, we demonstrated that cancer-associated fibroblasts (CAFs) in P-LN were associated with immune regulation, while CAFs in tumor underwent EMT. Our findings offered novel insights into the mechanisms of research, diagnosis, and therapy of CC metastasis.

## Introduction

Cervical cancer (CC) remains one of the most frequent female malignant tumors worldwide. The invasion and metastasis of CC in different organs represent the major challenges to its poor prognosis (1–3). The pelvic lymph node is the main target of CC metastasis, and tumor cells can invade the lymphatic system (3). In the process of metastasis, tumor cells evolve fitness and show tropism toward specific organs by activating specific genes and pathways, and tumor-recruited immune cells also play a pivotal role in promoting the progress of metastasis (4, 5). Nevertheless, the molecular mechanisms of cervical cancer invasion and lymph node metastasis have not been elucidated.

Advances in single-cell RNA sequencing (scRNA-seq) technology have enabled accurate and in-depth studies of cellular heterogeneity and the tumor microenvironment (TME) (6, 7). Recently, some studies have provided new insights into the characteristics of metastatic tumors (8–10). In the specific context of CC, our previous study has established the transcriptional profiles of normal and tumor cells in CC using scRNA-seq methods (11). Meanwhile, we also elucidated the characteristics of CC TME, from precancerous lesions to invasive tumors, and then to metastatic lymph nodes. However, the mechanism of malignant cell metastasis is still lacking (11, 12). Previous scRNA-seq studies have reported that metastatic cancer cells highly express genes related to epithelial–mesenchymal transition (EMT), oxidative stress, the proteasome, and cancer stem cell biomarkers (13–15). In addition, cancer-associated fibroblasts (CAFs) are also involved in the progression of tumor metastasis (16, 17). However, when CC metastatic tumors encounter the immune system in regional lymph nodes, their interactions have not been fully determined. This may be a dynamic situation involving persistent mutation and proliferative events in the surviving metastatic cells and the immune system. In the process, the immune system becomes “tolerant” and fails to further attack the metastatic cells as they grow in place and then continue to other regional lymph nodes.

In order to elucidate the transcriptional characteristics of tumor cells, immune cells, and stromal cells in metastatic LN and primary tumors, we performed an scRNA-seq analysis in the primary tumor, metastatic LNs, and normal LNs. Our analysis indicated that tumor cells from metastatic LN exhibited a high enrichment of cell cycle and immune regulation-related signaling pathways compared with tumor cells from primary tumors. By comparing the immune characteristics of normal LN and primary tumors, we found an early activated TME in metastatic LN. We then confirmed that MRC1, as a marker of macrophages in metastatic LN, might be used as a target of tumor therapy. Meanwhile, we found that CAFs in metastatic LN could regulate the immune response, which might be related to the immune escape of tumor cells. This study provided a comprehensive and rich landscape of the human CC transcriptome and provided molecular insights into CC metastasis.

## Methods

### Enrollment of patients

A total of four patients were recruited for this study after it was approved by the ethics committee of our hospital. Written informed consent was signed for all experiments involving patients. All patients did not receive any treatment before surgery. Moreover, the histological type of any patient was confirmed by standard histopathology. Only cervical squamous cell carcinoma (SCC) was included. Fresh samples were obtained at the time of surgery and confirmed by intraoperative frozen pathology. All tissues were then immediately placed in tissue protection fluid.

### Tissue processing for single-cell suspension

All tissue samples from the primary tumor and metastatic lymph node were washed with phosphate-buffered saline (PBS) three times on ice and then minced into small pieces. Subsequently, tissue was digested in a 2-ml dissociation medium containing 0.5 mg/ml collagenase IV (Sigma-Aldrich; St. Louis, MO) and 1 mg/ml DNAse I (Sigma-Aldrich; St. Louis, MO) in RPMI-1640 (ThermoFisher Scientific) following the manufacturer’s instructions. Briefly, the samples were then incubated at 37°C for 30 min, rotating manually every 10 min. The neutralization reaction was then supplemented with 1 ml of cold RPMI-1640 containing 10% fetal bovine serum (FBS, ThermoFisher Scientific). A 40-µm nylon mesh (ThermoFisher Scientific) was used to separate cells from impurities after digestion. Samples were centrifuged at 300×*g* for 5 min, and cell pellets were resuspended in 1 ml of PBS. The supernatant was discarded. Cell suspensions were counted to determine cell concentration and viability.

### Single-cell sequencing

The density of cell suspensions was adjusted in 1 × 10^5^ cells/ml in PBS and then loaded on a Chromium Single Cell Instrument (10× Genomics) to produce single-cell gel bead-in-emulsion (GEMs). A single-cell RNA sequence library was estimated using version 1 Chromium Single-Cell 30 Library, Gel Bead & Multiplex Kit (10× Genomics) according to the manufacturer’s instructions. The resulting scRNA-seq libraries were sequenced on an Illumina HiSeq ×10 instrument with 150-bp paired-end reads. Cell Ranger (version 3.0.1) was used with default parameters to perform sample demultiplexing, barcode processing, and single-cell gene unique molecular index counting (https://software.10xgenomics.com/single-cell/overview/welcome) (18).

### QC and cell- type identification

Seurat (version 3.0.1) was used for the procession QC (19). Cells with <200 unique molecular identifiers (UMIs) or mitochondrion-derived UMI counts of >10% in a single cell were considered low-quality cells and removed. The top 30 principal components and the first 2,000 variable genes were used in this process. The inflow of UMI count and the percentage of mitochondrion-derived UMI counts were regressed by the ScaleData function. Subsequently, Seurat’s “find clusters” function was used to identify the main cell clusters. The Louvain clustering algorithm embedded in Seurat software was used for clustering, and the t-distributed stochastic neighbor embedding (tSNE) method was used to visualize the clustering results. For any cell cluster, it was mainly identified because of the differences in cell cycle and did not participate in downstream analysis. To accurately annotate cell types, we manually collated genetic markers for each cell type. In particular, most of the markers used to distinguish different cell types were retrieved from the Cell Markers database (https://www.labome.com/method/Cell-Markers.html) (20). Other marker genes came from published papers.

### DEGs and GSVA

The specific markers for each cluster were identified by performing the FindAllMarkers function on the standardized expression data in the Seurat software package (only.pos = T, min.pct = 0.25) (21). Genes with adjusted *p*-value < 0.05 were considered statistically significant for KEGG and GO enrichment analyses. The ClusterProfiler package (version 3.14.3) was used to enrich and analyze cluster-specific biomarker genes (22). GSEA was performed with MSigDB gene sets to determine the differential pathways (23). The full gene lists of T-cell signatures (including the cytotoxic, exhausted, regulatory, naive, and costimulatory activity of T cells) were extracted from the published report by Chung et al. (24).

### Trajectory analysis

Trajectory analysis was performed using Monocle 2 (version 2.8.0) (25). We then performed differential gene expression analysis using the differential gene test function to identify significant genes (BH-corrected *p* < 0.01). The cellular ordering of these genes was done in an unsupervised manner. Trajectory construction was then performed after dimensionality reduction and cell sorting with default parameters.

### Calculation of functional module scores

To evaluate the potential functionality of the cell cluster of interest, we calculated the scores of functional modules in the cell cluster using the AddModuleScore function in Seurat. The average expression levels of the corresponding cluster were subtracted by controlling the aggregated expression of the feature set. All analyzed genes were binned based on average expression, and control characteristics were randomly selected from each bin.

### CNV analysis

Copy number variation (CNV) analysis based on scRNA-seq CNV was estimated by using the R package inferCNV (https://github.com/broadinstitute/inferCNV; v1.6.0), which sorts the genes according to their chromosomal location and applies a moving average to the relative expression values. For the grouping information of cells, one is by sample, and the other is by cluster, or cell type. An average value of CNV was estimated in nonoverlapping genomic regions. Average CNV values were rounded to the closest integers.

### Correlation to public datasets

To assess the prognostic effect of individual genes or each set of signature genes, the TCGA-CESC data were used. The patient cohorts were divided into high- and low-expression groups according to the median value of the normalized mean expression of strong marker genes (logFC>2). Kaplan–Meier survival curves were generated in R-4.0.3 with the R package “survminer”.

### Statistical analysis

Statistical analysis was performed using SPSS 20.0 (Chicago, IL, USA), and statistical significance was determined with a *t*-test. The *p*-values were calculated. Unless specifically stated, *p* < 0.05 was considered statistically significant.

## Results

### Single-cell transcriptomic analysis of primary tumor and metastatic lymph node

**Figure 1:**
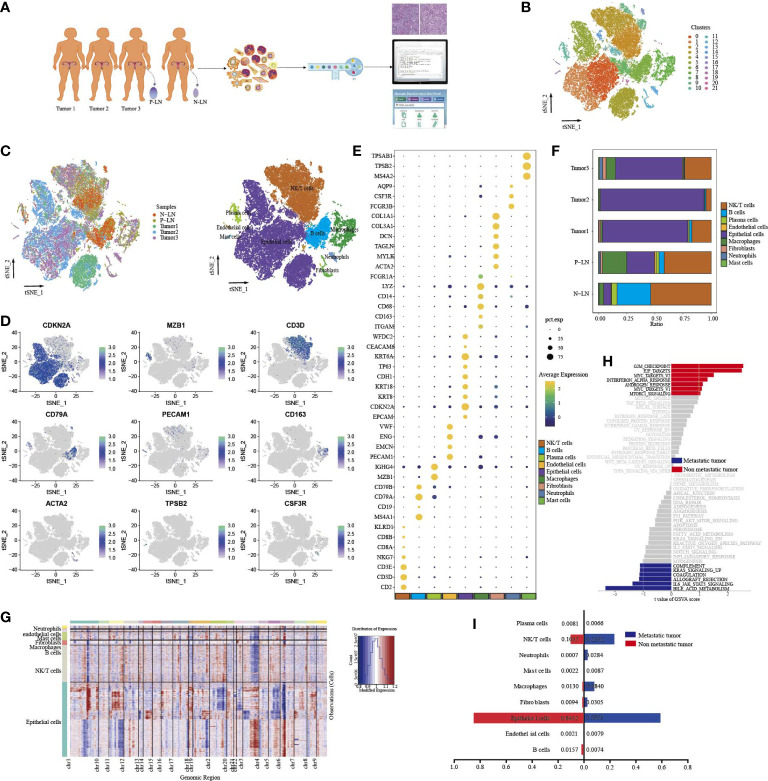
Single-cell transcriptome profiles of CC primary tumor and metastatic lymph node. Workflow depicting the collection and processing of samples for scRNA-seq analysis **(A)**. tSNE of all cells profiled here, with each cell color-coded for clusters **(B)**. tSNE of all cells profiled here, with each cell color-coded for (left to right) the corresponding samples and the cell types **(C)**. tSNE of representative genes for each cell type **(D)**. Dot plot of significant marker genes for each cell type **(E)**. The proportion of each cell type in five samples **(F)**. Heatmap showing large-scale CNVs of each cell type **(G)**. GSVA of hallmark gene sets between metastatic tumor (tumor 3) and non-metastatic tumors (tumors 1 and 2) **(H)**. The difference in cell type proportion between metastatic tumors (tumor 3) and non-metastatic tumors (tumors 1 and 2) **(I)**.

To investigate the cellular diversity and molecular signatures of CC metastasis, we performed 10× Genomics scRNA-seq analysis on three tumor samples (tumors 1, 2, and 3) and one positive-lymph node (P-LN) and one negative-lymph node (N-LN) from four patients. Tumor 3 and P-LN were from the same patient. Tumors 1 and 2 from two patients without LN metastasis were defined as nonmetastatic tumors, while tumor 3 from one patient with LN metastasis was defined as a metastatic tumor (**Figure 1A**). After quality control, we obtained 51,507 cells, including 31,988 from the primary tumor, 9,065 from P-LN, and 10,454 from N-LN tissue. After gene expression normalization, we performed dimensionality reduction and clustering using principal component analysis (PCA) and tSNE, respectively. All cells were divided into 22 clusters (clusters 0–21) (**Figure 1B**). Through the expression of typical marker genes, all clusters could be divided into nine cell lineages by canonical marker gene expression (**Figures 1C–E**; **Supplementary Figures S1A, B**): epithelial cells (27,641 cells, 53.66%, marked with EPCAM, CDKN2A, and CDH1); macrophages (3,418 cells, 6.64%, marked with CD163, CD68, and CD14); mast cells (183 cells, 0.36%, marked with TPSB2, MS4A2, and TPSAB1); B cells (3,963 cells, 7.69%, marked with CD19, CD79A, and CD79B); fibroblasts (685 cells, 1.33%, marked with ACTA2, TAGLN, and COL1A2); endothelial cells (305 cells, 0.59%, marked with ENG, PECAM1, and EMCN); neutrophils (429 cells, 0.83%, marked with FCGR3B, AQP9, and CSF3R); and plasma cells (979, 1.90%, marked with MZB1 and IGHG4). In addition, cells in C2, C3, C9, C12, and C15 highly expressed CD3E, CD3D and NKG7, so they were defined as NK/T cells (13,904 cells, 26.99%) (**Supplementary Figure S1A**). The proportion of each cell lineage varied among different samples (**Figure 1F**; **Supplementary Figure S1C**). To distinguish cancer cells from other cell types, we determined the main copy number aberration (CNA) events in each cell type according to their transcriptomic profile (**Figure 1G**). Remarkably, tumor cells presented a high level of CNV and had prominent molecular intertumor heterogeneity.

In order to explore the characteristic between metastatic tumors and non-metastatic tumors, a functional analysis of hallmark gene sets was performed (**Figure 1H**). The result showed that nonmetastatic tumors exhibited high enrichment of cell-cycle signaling pathways, such as G2M checkpoint, E2F targets, MYC targets V2, and mitotic signal, while inflammatory-related signaling pathways, such as IL6_JAK_STAT3 signaling, coagulation, allograft_rejection, compliment, and inflammatory response were upregulated in metastatic tumors. We also found that metastatic tumors had higher immune cell infiltration, such as macrophages, neutrophils, and NK/T cells, but lower epithelial cells than nonmetastatic tumors (**Figure 1I**). These results indicated that once tumor metastasis occurs, tumor cells undergo a series of functional changes to adapt to TME, which is conducive to the immune escape of tumor cells.

### Characteristics of epithelial cells in primary tumors and metastatic lymph nodes

**Figure 2:**
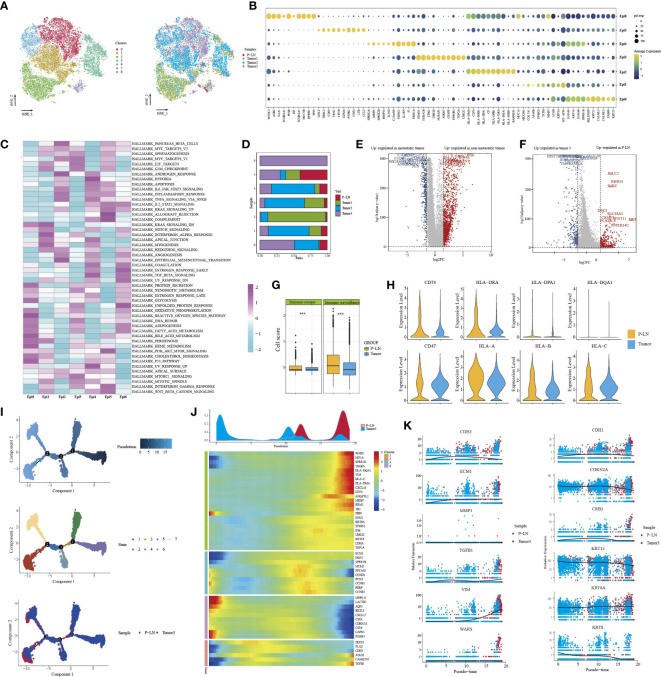
Characteristics of epithelial cells in the primary tumor and metastatic lymph node. tSNE of epithelial cells, with each cell color-coded for (left to right) the seven clusters of all cells and the corresponding samples **(A)**. Dot plot of representative genes in each cluster **(B)**. Differences in pathway activity (scored per cell by GSVA) in seven tumor cell clusters **(C)**. The proportion of each cell cluster in four samples **(D)**. Volcano plot showing differentially expressed genes of epithelial cells between metastatic tumors (tumor 3) and non-metastatic tumors (tumors 1 and 2) **(E)**. Volcano plot showing differentially expressed genes of epithelial cells between metastatic LN (P-LN) and primary tumor (tumor 3) **(F)**. Boxplots showing the differences of immune escape and immune surveillance score between P-LN and tumor (***P < 0.001) **(G)**. Violin plot showing the differences of significant genes between metastatic LN (P-LN) and primary tumor (tumor 3) **(H)**. Pseudotime analysis of epithelial cells predicated by Monocle 2. Each point corresponds to a single cell, and the color of the dot is distinguished by different times (up), different states (middle), and different samples (down) **(I)**. Heatmap showing dynamic changes in gene expression along the pseudotime (lower panel). Distribution of epithelial cells during the transition along with the pseudotime. Samples were labeled by colors (upper panel) **(J)**. Two-dimensional plots showing the expression scores of genes related to EMT along with the pseudotime **(K)**.

We identified seven clusters (Epi 0–6) following the reclustering of 27,641 epithelial cells (**Figure 2A**). All clusters expressed different epithelial markers, and each subpopulation had specific markers and different functional states (**Figures 2B, C**; **Supplementary Figure S2**). For example, cells in cluster 0 expressed high levels of epithelial cell markers (KRT15, FABP4, and KRT16) and were associated with high enrichment of epithelial cell differentiation, regulation of peptidase activity, and cell–cell adhesion. Cells in cluster 2 were characterized by high expression of HLA-DRB1, HLA-DRA, and CD74. GO analysis of upregulated genes showed that cytoplasmic translation and antigen processing and presentation were highly enriched. Cluster 4 expressed a high level of cell-cycle genes, such as MKI67, TOP2A, and UBE2C, and functional analysis showed high enrichment of cell division, regulation of cell cycle, and regulation of chromosome segregation. We then compared the percentage of cell subpopulations in different samples (**Figure 2D**). Importantly, the majority of cells in cluster 5 were from P-LN and tumor 3, and they exhibited high expression of GNLY, CD69, and GZMA, indicating that they were closely related to immune cells.

Tumor metastasis occurs when cancer cells leave the primary tumor and seed metastasis in regional lymph nodes or distant sites. Before metastasis, tumor cells themselves exhibit cellular changes involved in this complex process to escape the attack of immune cells (26). We compared the transcriptional characteristics of tumor cells in nonmetastatic tumors (tumors 1 and 2) and metastatic tumors (tumor 3). The results showed that tumor cells from nonmetastatic tumors expressed high levels of MMP1, KRT1, and MMP13 and low levels of MGST1, FAM133A, and FMO3 (**Figure 2E**; **Supplementary Table S1**). GO analysis of DEGs (|log2FC|>2) showed that cell adhesion, extracellular matrix organization, and vasculature development were highly enriched (**Supplementary Figure S3A**). However, epithelial cells in metastatic tumors were associated with high enrichment of immune regulation-related signals, such as granulocyte chemotaxis, leukocyte-mediated immunity, and granulocyte action (**Supplementary Figure S3B**).

Although many studies have reported that tumor cells in primary tissue and metastatic LN share some similar transcriptional characteristics, these characteristics may change during the molecular evolution of tumor cell metastasis (27). Primary tumor, if available, does not always provide sufficient information to understand the characteristics of metastasis LN. Therefore, we compared the transcriptional differences of tumor cells in primary tumors and metastatic LN. First, we obtained 957, 982, and 220 DEGs between P-LN vs. tumor 1, P-LN vs. tumor 2, and P-LN vs. tumor 3, respectively (Log2FC>2 and p_val_adj<0.05) (**Supplementary Figure S3C**; **Supplementary Table S1**). A Venn diagram showed 120 significantly overlapping upregulated genes in P-LN, and GO analysis showed that various immune-related signaling pathways were highly enriched, such as lymphocyte activation, positive regulation of immune response, and immune effector process, which represented a common function (**Supplementary Figure S3D**). Importantly, tumor 3 and P-LN came from the same patient, so we focused on the analysis of tumor cell function between tumor 3 and the P-LN sample (**Figure 2F**). The results showed that tumor cells in metastatic LN expressed high levels of IGLC2, IGHG4, and IGKC, and GO analysis showed various immune-related signaling pathways (**Supplementary Figure S3E**). However, tumor cells in tumor 3 expressed S100A7, FABP4, and KRT14, and GO analysis showed that complement activation, epithelial cell differentiation, and negative regulation of growth were highly enriched (**Supplementary Figure S3F**). We also observed that tumor cells in P-LN presented higher immune surveillance and escape than those in tumor 3 (**Figure 2G**). Among the genes related to immune escape, we observed an increase in the expression of CD47 and MHC-II molecular in P-LN, indicating that DC maturation and antigen presentation might be inhibited, as well as resistance to macrophage targeting, accompanied by a decrease in the release of new antigens. Similarly, we observed increased expression levels of HLA/B/C in P-LN, indicating an increased resistance to NK-mediated cell death (**Figure 2H**). We then evaluated the metabolism level of tumor cells in each cluster (**Supplementary Figure S4**). The results showed that each cluster had specific enrichments of metabolism-related signaling pathways. However, the metastatic lymph node had a lower metabolic level compared with that in the tumor.

In order to understand the functional difference of tumor cells between the primary lesion and metastatic LN, we then utilized Monocle 2 to perform a pseudotime analysis to explore the cell transitions and dynamics of tumor cells. We observed that cells in the tumor and metastatic LN were located at both ends of the trajectory and formed a continuum but had different expression characteristics (**Figures 2I, J**). Compared to cells in tumor 3 that expressed increased epithelial markers like CDKN2A, CRB3, KRT13, and KRT6, tumor cells in metastatic LN exhibited high expression of mesenchymal markers like CDH3, ECM1, TGFB1, WARS, and VIM, indicating that tumor metastasis was related to EMT (**Figure 2K**; **Supplementary Table S1**). EMT and its reverse mesenchymal-to-epithelial transition (MET) are considered to play a crucial role in tumor metastasis (28). It has been reported that tumor cells with EMT have strong motility and tend to metastasize (29). According to TCGA data, we found that high expression of classical mesenchymal markers (e.g., CDH2, TGFBI, and ITGB1) had a worse prognosis, while the expression of epithelial markers (e.g., CDH1, CRB3, and DSP) had no effect on the survival, indicating that mesenchymal markers could be used as a predictor of CC metastasis (**Supplementary Figure S5**).

### Immune characteristics of NK/T cells between the primary lesion and metastatic lymph node

**Figure 3:**
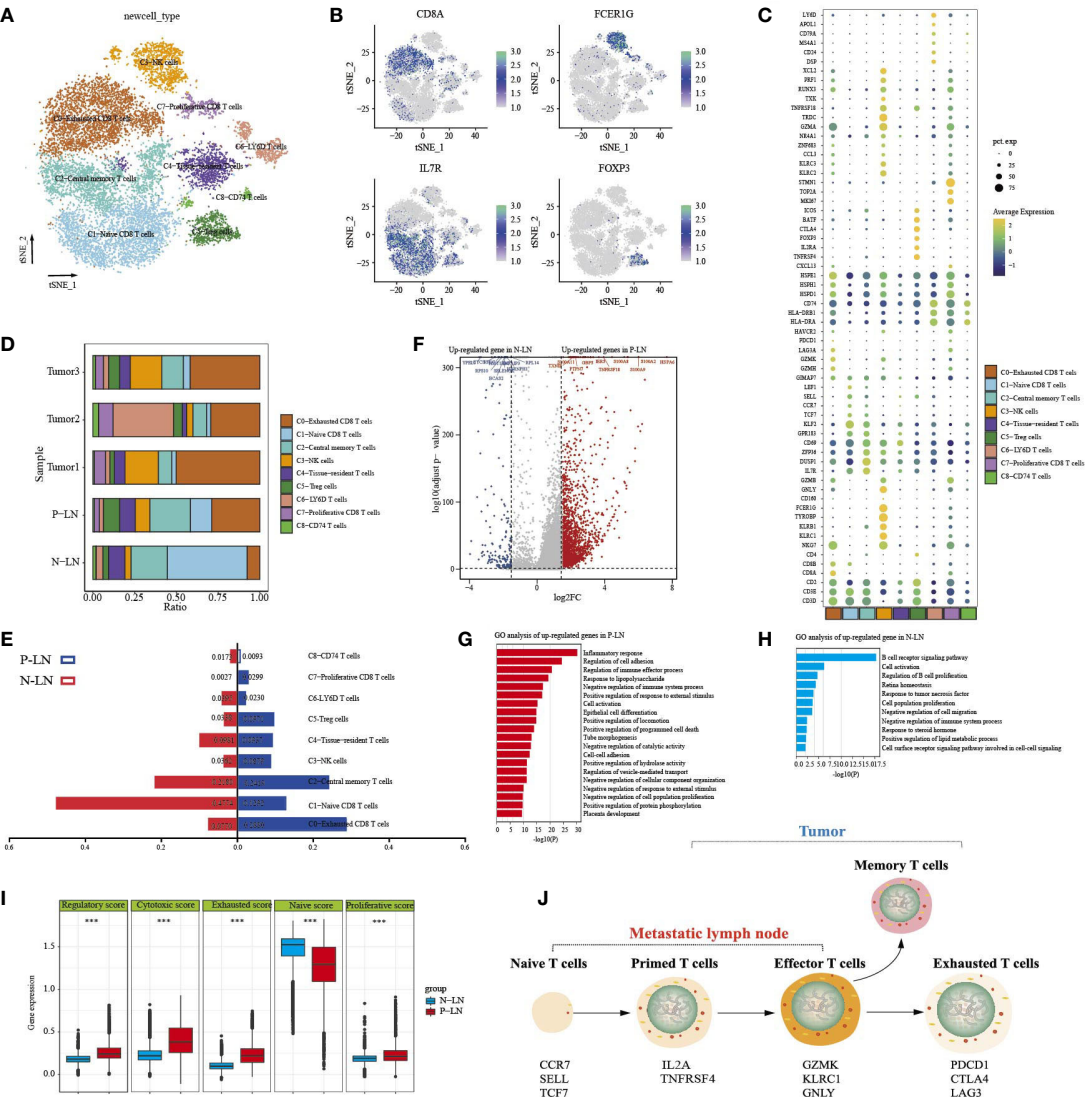
Analysis of NK/T- cell transition status in primary tumors and metastatic lymph nodes. tSNE of nine T/NK cell clusters, with each cell color-coded for nine cell types **(A)**. tSNE of marker genes for each cell type **(B)**. Dot plot of significant representative genes in nine cell types **(C)**. The proportion of each T/NK subpopulation in five samples **(D)**. The difference of each subpopulation proportion between P-LN and N-LN **(E)**. Volcano plot showing differentially expressed genes of NK/T cells between P-LN and N-LN **(F)**. GO analysis of upregulated DEGs of all NK/T cells in P-LN **(G)**. GO analysis of upregulated DEGs of all NK/T cells in N-LN **(H)**. Comparison of a regulatory, cytotoxic, exhausted, naive, and proliferative score of all NK/T cells between P-LN and N-LN (***P < 0.001) **(I)**. Development characteristics of NK/T cells in metastatic LN and primary tumor **(J)**.

To better understand the transcriptional heterogeneity in tumor-infiltrating T lymphocytes (TILs), we identified T- cell clusters that expressed known T- cell markers (CD3D and CD3E). Reclustering of 13,904 NK/T cells revealed nine clusters (**Figure 3A**), which were designated as CD8 T cells (CD8A and CD8B; clusters 0, 1, and 7), natural killer T cells (KLRC1, cluster 3), regulatory CD4 T cells (FOXP3 and CD4, cluster 5), central-memory T cells (IL7R, and DUSP1, cluster 2), antigen-presentation T cells (CD74, and HLA-DRA, clusters 6 and 8), and tissue-resident T cells (CD69 and GPR183, cluster 4) (**Figures 3B, C**). We then further analyzed the differences in CD8 T- cell clusters. C0-exhausted CD8 T cells expressed high levels of immune checkpoint genes (such as LAG3, PDCD1, and HAVCR2). Importantly, these cells also had high expression levels of NKG7, GZMK, and GZMH, indicating the coexistence of cytotoxicity and exhausted cells. C1-naive CD8 T cells exhibited high expression of KLF2, CCR7, SELL, and LEF1 and were defined as naive CD8 T cells. C7-proliferative CD8 T cells expressed cell-cycle genes, such as MKI67, TOP2A, and STMN1, which were defined as proliferative CD8 T cells. C5-Treg cells exhibited high expression of CD4, FOXP3, and IL2RA, as well as immune checkpoint genes (CTLA4, and BATF), indicating an immunosuppressive cell type. In addition, we found that both clusters 6 and 8 exhibited high expression of CD74, HLA-DRB1, and HLA-DRA, representing the type of antigen-presentation cell types. Cluster 6 was associated with the high expression of CD79, CD24, LY6D, and MS4A1, indicating that C6-LY6D T cells were closely related to other immune cells (**Figure 3C**).

We then evaluated the distribution of immune cells (**Figure 3D**). A remarkable feature of normal LNs was the high infiltration of naive T cells, which reflected the basic function of LNs. LN is a dynamic organ that can be dramatically remodeled under pathological conditions, such as inflammation or cancer. In metastatic LN, the number of central memory T cells, exhausted CD8 T cells with cytotoxicity, and Treg cells increased, while the number of naive cells decreased (**Figure 3E**). We then performed a differential analysis of all cells between P-LN and N-LN (**Figure 3F**; **Supplementary Table S1**). The results showed that immune cells in P-LN were associated with various signaling pathways, including inflammatory response, cell activation, and immune effector process (**Figure 3G**), whereas immune cells in N-LN exhibited high enrichment of B-cell receptor signaling pathway, cell activation, and B-cell proliferation regulation (**Figure 3H**). Similarly, we also found that P-LN samples were associated with a higher regulatory score, cytotoxic score, exhausted score, and proliferative score but a lower naive score compared with N-LN (**Figure 3I**; **Supplementary Table S2**). These results indicated that in metastatic LN, T cells underwent the transition from naive T cells to effector T cells, or exhausted T cells, representing the activated stage of immune response (**Figure 3J**).

Our previous studies confirmed that CC had an immunosuppressive TME. However, data on the difference in TME between metastatic and non-metastatic tumors were limited. In the present study, we found that metastatic tumors had a high percentage of Treg cells and central memory T cells but low proliferative CD8 T cells and antigen-presentation cells (**Supplementary Figure S6A**). We then found that adaptive immune response, humoral immune response, regulation of cell activation, and immune effector process were highly enriched in T cells from the metastatic tumor (**Figures S6B, C**). However, T cells from non-metastatic tumors were associated with high enrichment of extracellular matrix organization, cell motility, and cell junction organization (**Figure S6D**).

### The characteristics of macrophages in primary lesion and metastatic lymph node

**Figure 4:**
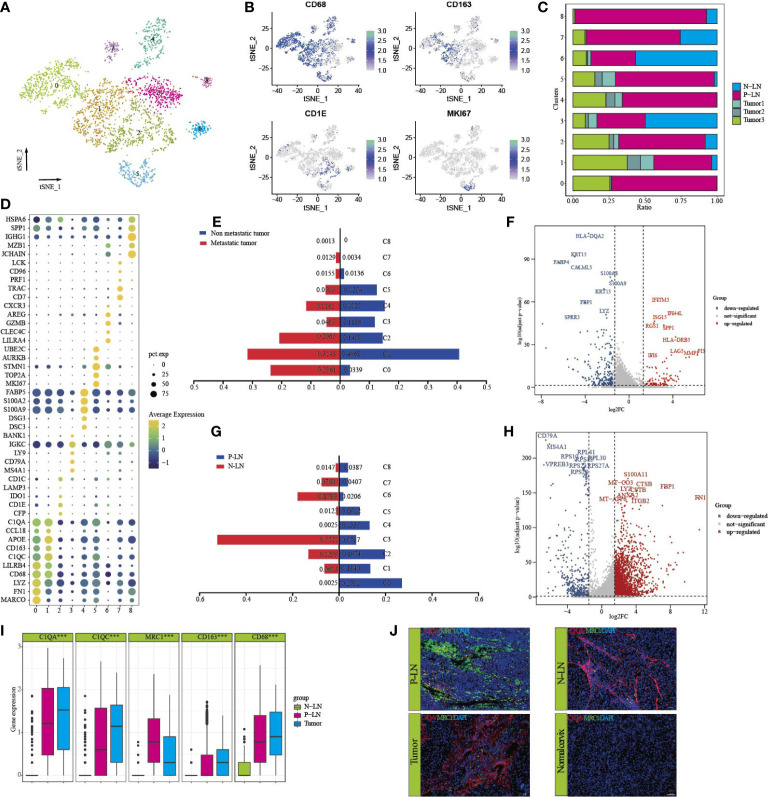
Characteristics of macrophages in primary tumor and metastatic lymph node. tSNE of macrophages, with each cell color-coded for nine clusters of all cells (C0–C8) **(A)**. tSNE of marker genes for each cluster **(B)**. The proportion of each sample in nine clusters **(C)**. Dot plot of significant representative genes in nine clusters **(D)**. The difference in each cluster proportion of macrophages between metastatic tumors (tumor 3) and non-metastatic tumors (tumors 1 and 2) **(E)**. Volcano plot showing differentially expressed genes of macrophages between metastatic tumor (tumor 3) and non-metastatic tumors (tumors 1 and 2) **(F)**. The difference of each cluster proportion of macrophages between P-LN and N-LN **(G)**. Volcano plot showing differentially expressed genes of macrophages between P-LN and N-LN **(H)**. Bar plots showing the expression of C1QA, C1QC, MRC1, CD163, and CD68 in P-LN, N-LN, and primary tumor (^***^ *p* < 0.001) **(I)**. Immunofluorescence staining of significant markers in P-LN, N-LN, primary tumor, and adjacent normal tissue blue = DAPI, green = MRC1, red = C1QA **(J)**.

Reclustering of 3,418 macrophages identified nine cell types (**Figure 4A**). Each subpopulation exhibited specific markers and functional status. Most clusters had high expression levels of CD163 and CD68 (**Figure 4B**), and only C2 expressed high levels of CD1C and CD1E. Importantly, we found that most macrophages were from P-LN and tumor 3 samples, indicating the potential role of macrophages in promoting tumor metastasis (**Figure 4C**). We found that each cluster represented different functional enrichment (**Figure 4D**; **Supplementary Figure S7**).

Our previous study confirmed that the tumor had a dominant M2-like signature and represented an immunosuppressive TME. However, there is limited data on the difference in macrophage types between metastatic and nonmetastatic tumors. We found that the number of macrophages of C0, C2, and C7 in metastatic tumors was higher, but the number of macrophages of C1, C3, and C5 was lower than that in nonmetastatic tumors (**Figure 4E**). Differential analysis showed that IFITM3, IFI44L, ISG15, SPP1, LAG3, and HLA-DRB5 were highly expressed in non-metastatic tumors (**Figure 4F**; **Supplementary Table S1**). GO analysis showed high enrichment of cell differentiation, the collagen metabolic process, and endopeptidase activity (**Supplementary Figure S8A**). However, macrophages in metastatic tumors had high expression of HLA-DQA2, KRT15, S100A8, and S100A9, and GO analysis showed that lipid catabolic process, myeloid leukocyte migration, and the regulation of cell activation were highly enriched (**Supplementary Figure S8B**; **Supplementary Table S1**). In addition, the metastatic tumor had a higher M2-signature score but a similar M1-signature score to the non-metastatic tumor (**Supplementary Figure S8C**; **Supplementary Table S3**). These results indicated that macrophages supported tumor progression, invasion, and metastasis by promoting the immunosuppression of tumor cells.

We also compared the differences in macrophages between P-LN and N-LN. We found that macrophages in C0, C1, C4, and C5 were the main cell types in P-LN, while C3, C6, and C7 were the main types in N-LN (**Figure 4G**). Differentially, analysis showed high expression of FN1, FBP1, S100A11, CTSB, and ITGB2 (**Figure 4H**) in P-LN, and GO analysis confirmed high enrichment of the inflammatory response, cell motility, and cellular response cytokine stimuli (**Supplementary Figure S8D**). However, macrophages in N-PN were involved in the regulation of B cells, such as B-cell activation, leukocyte activation, and regulation of B-cell proliferation (**Supplementary Figure S8E**). By calculating the M1 and M2 signature scores using related gene sets, we observed that P-LN had a higher M2 signature score and M1 signature score than that in N-LN, which represented an immunosuppressive TME (**Supplementary Figure S8F**; **Supplementary Table S3**).

In our previous study, we demonstrated that macrophages defined as Ma-C1QA highly expressed MRC1, APOC1, GPNMB, and CTSD and could also be defined as M2-like macrophages. Ma-THBS1 highly expressed VCAN, TIMP1, IL1B, EREG, and FCN1 and could be defined as M1-like macrophages. Importantly, most Ma-C1QA were from tumors or metastatic LNs, but according to our previous data, we cannot distinguish the exact differences in macrophages between the primary lesion and metastatic LN. By analyzing the differential genes between P-LN and tumor 3, we found that macrophages in P-LN exhibited high expression of IGHG, IGLC2, IGKC, IGHG1, CD52, CD1B, MRC1, SPP1, and LGALS2, whereas the primary tumor exhibited high express ion of SDS, FABP4, SPRR3, CLDN4, MDK, and IFI27 (**Figure 4D**; **Supplementary Table S1**). MRC1 (CD206) is a scavenger receptor that contributes to multiple cellular functions and is usually highly expressed in M2-like macrophages (30). Gao et al. recently reported that the infiltration level of MRC1+ CCL18+ macrophages was elevated in the metastatic lesion (31). In this study, we found that C1QA and C1QC were highly expressed in both tumors and metastatic LN, while MRC1 was only highly expressed in metastatic LN, indicating that MRC1 could be used as a marker to predicate tumor metastasis. We then defined tumor-derived macrophages as C1QA+MRC1^low^ macrophages and LN-derived macrophages as C1QA+MRC1^high^ macrophages (**Figure 4I**). Immunofluorescence results showed that C1QA expression in tumor, P-LN, and N-LN was higher, while MRC1 in P-LN was higher than that in tumor and N-LN (**Figure 4J**).

### Lymph node-derived CAFs promoted immune response

**Figure 5:**
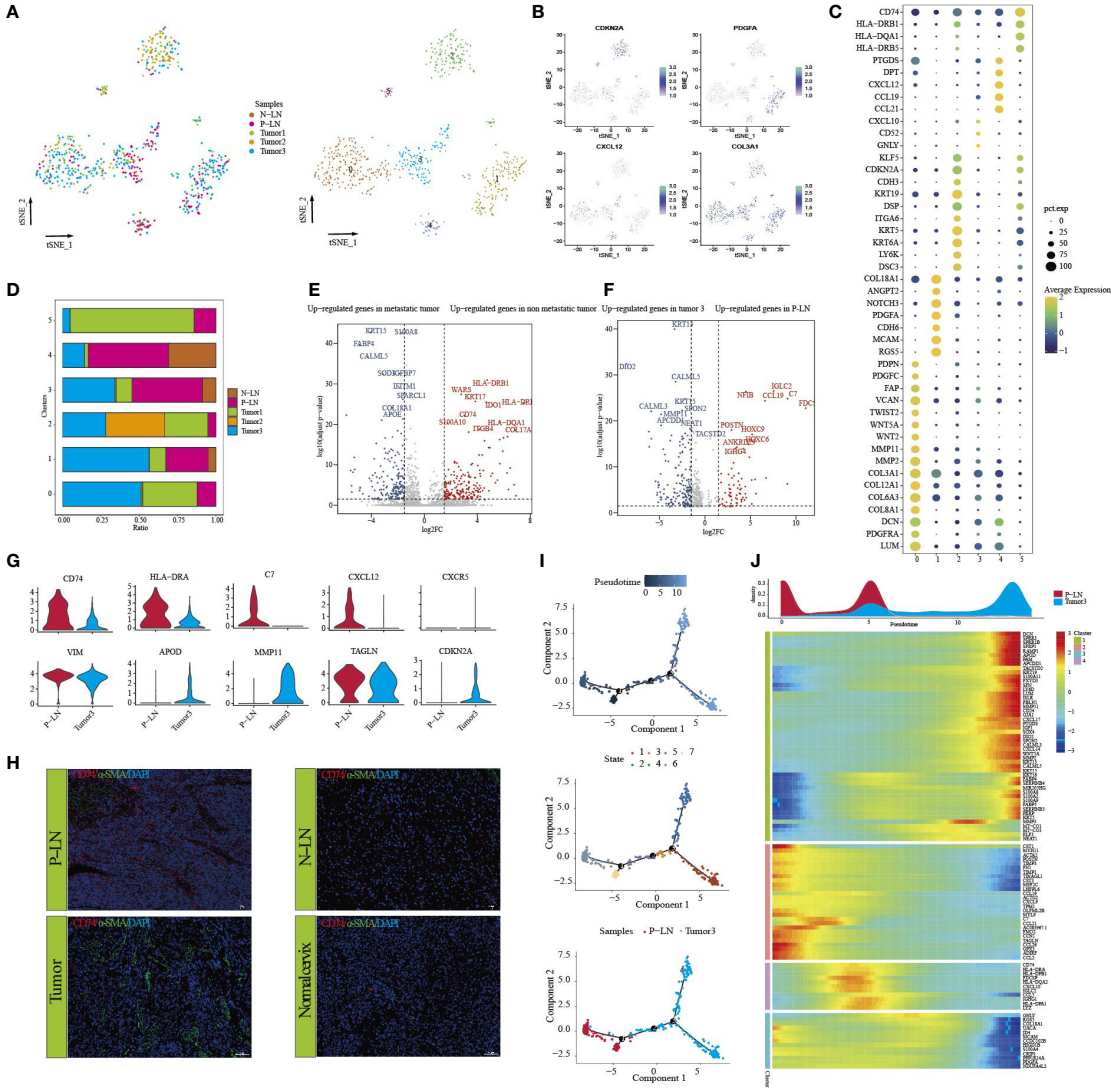
Analysis of CAF transition states in primary tumor and metastatic lymph node. tSNE of CAFs, with each cell color-coded for (left to right) the corresponding patient and six clusters of all cells (C0–C5) **(A)**. tSNE of marker genes for each cluster **(B)**. Dot plot of significant representative genes in six clusters **(C)**. The proportion of each sample in five samples **(D)**. Volcano plot showing differentially expressed genes of CAFs between metastatic tumor (tumor 3) and non-metastatic tumors (tumors 1 and 2) **(E)**. Volcano plot showing differentially expressed genes of CAFs between P-LN and tumor 3 **(F)**. Violin plots showing the expression difference of significant genes between P-LN and tumor 3 **(G)**. Immunofluorescence staining of significant markers in P-LN, N-LN, primary tumor, and adjacent normal tissue. Blue = DAPI, green = α-SMA, and red = CD74 **(H)**. Pseudotime analysis of CAFs predicated by Monocle 2. Each point corresponds to a single cell, and the color of the dot is distinguished by different times (up), different states (middle), and different samples (down) **(I)**. Heatmap showing dynamic changes in gene expression along the pseudotime (lower panel). Distribution of CAFs during the transition along with the pseudotime. Samples were labeled by colors (upper panel) **(J)**.

CAFs have long been considered the representative of heterogeneous populations, but the extent of heterogeneity has hitherto remained unexplored because CAF phenotypes are considered highly context-dependent and unstable in culture (32). In our previous study, we found that the majority of fibroblasts came from normal tissue, so it is difficult to evaluate the function of fibroblasts according to our previous data. In this study, 685 fibroblasts were obtained from tumors and metastatic LN, and they were defined as CAFs. Subclustering revealed six distinct subtypes (**Figure 5A**). Each cluster expressed specific markers (**Figures 5B, C**) and functional states (**Supplementary Figure S9**). For example, CAFs in cluster 2 expressed high levels of CDKN2A KRT19, DSP, KRT5, and KRT6A, indicating that these cells may be associated with EMT. Clusters 1, 3, and 4 had a high percentage of macrophages from tumor 3 and P-LN (**Figure 5D**; **Supplementary Table S1**), indicating that these cells were associated with tumor metastasis. We then analyzed the differential expression of CAFs between metastatic tumors and non-metastatic tumors. The results showed that CAFs in metastatic tumors expressed high levels of KRT15, FABP4, S100A8, and COL18A1 and were associated with high enrichment of vasculature development, cell population proliferation, and epithelial cell differentiation. However, CAFs in primary tumors expressed high expression of COL17A1, MMP10, MMP13, and CD74 (**Figure 5E**; **Supplementary Table S1**). GO analysis showed high antigen processing, skin development, tissue morphogenesis, and positive regulation of cell motility (**Supplementary Figures S10A, B**). These results indicated that CAFs from metastatic tumors could improve the proliferative and differentiation of tumor cells and reduce the activation of the immune response.

We also compared CAF differences between metastatic LN and primary tumor (P-LN vs. tumor 3). The results showed that CAFs in P-LNs were associated with the high expression of NFIB, IGLC2, IGHG4, C7, and CCL19, while CAFs in tumor 3 had high expression of DIO2, LGALS1, WNT5A, NEAT1, and MMP11 in the tumor (**Figure 5F**; **Supplementary Table S1**). Functional analysis showed that P-LN in CAFs was associated with the regulation of cell activation, immune response, and regulation of the ERK1 and ERK2 cascades. However, tumor-derived CAFs exhibited high enrichment of epithelial cell differentiation, tube morphogenesis, and gland development (**Supplementary Figures S10C, D**). The differential analysis also confirmed the high expression of CD74, HLA-DRA, C7, CCL12, and CCR3 in CAFs from P-LN but lower expression of VIM, APOD, MMP11, TAGLN, and CDKN2A compared with that in the primary tumor (**Figure 5G**; **Supplementary Table S1**). Meanwhile, immunohistochemical analysis showed higher expression of CD74 in metastatic lymph nodes compared with that in the primary tumor (**Figure 5H**). These results further demonstrated that P-LN-derived CAFs participated in tumor immune regulation.

Finally, we evaluated the development of CAFs by performing pseudotime trajectory analysis using Monocle 2. All CAFs formed a continuum but had different expression features (**Figures 5I, J**). Importantly, CAFs in P-LN with high expression of immune-related genes, such as CD74, C7, C7, CCL12, and CD74, but CAFs in tumors had high expression of EMT-related genes, such as COL1A1, MMP11, TAGLN, and ACTA2 (**Figure 5J**). These outcomes further demonstrated that CAFs in metastatic LN played an immunomodulatory role.

### Molecular interactions of tumor cells with other cell types

**Figure 6:**
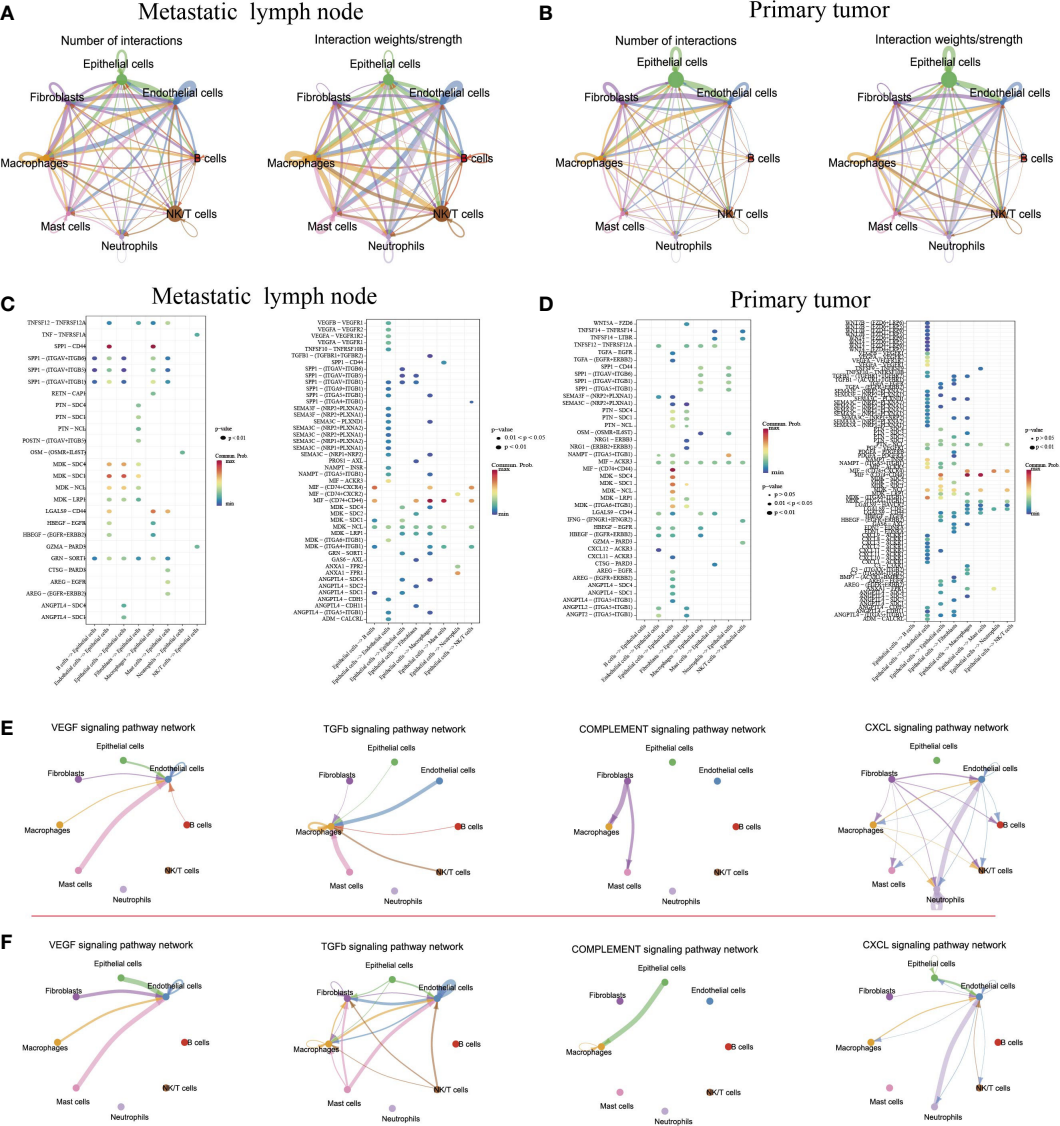
Intercellular and molecular interactions in primary tumor and metastatic lymph node. Chord diagram showing the number and strength of interactions in metastatic lymph nodes **(A)** and primary tumors **(B)**. Bubble diagram depicting the significant ligand–receptor interactions of epithelial cells with other cell types in metastatic lymph nodes **(C)** and primary tumors **(D)**. Significant signaling pathways of cell–cell connections in metastatic lymph nodes **(E)** and primary tumor **(F)**.

Understanding the interactions between tumor cells and immune cells and stromal cells is important to comprehending the mechanisms of cancer progression and metastasis. We then performed the cell–cell connections of tumor cells with other cell types in tumor and metastatic LN, respectively. The results showed that there were more cell–cell connections in metastatic LN than in tumors (**Figures 6A, B**). In metastatic LN, epithelial cells with endothelial cells, B cells, macrophages, and NK/T cells had more interactions, while epithelial cells in tumors had more connection with macrophages and endothelial cells. When we evaluated the effect of epithelial cells on other cell types, both groups shared some similar ligand–receptor pairs. However, epithelial cells in primary tumors could regulate the function of other cell types through several specific ligands, such as Wnt7B/4, VEGFA/B, PTN, PDGFA, LGALS9, and CXCL1/2/3/8/9/12 (**Figures 6C, D**). We then discussed the effects of other cell types on epithelial cells in tumor tissue, and the results indicated that immune cells could regulate epithelial cells through specific ligand–receptor pairs, such as TNFSF14-TNFRSF14, MIF-(CD74+CD44), IFNG-(FINGR1+IFNGR2), CXCL12-ACKR3, and CXCL11-ACKR3 (**Figures 6C, D**). Importantly, these cell–cell pairs were associated with the expression of immune checkpoint genes, indicating a better outcome for immunotherapy.

Finally, we explored the cell–cell connections for all cell types in several signaling pathways (**Figures 6E, F**). The results indicated that in the primary tumor, endothelial cells exhibited strong connections with mast cells, fibroblasts, and epithelial cells by the VEGFA signaling pathway and TGF-β signaling pathway. Meanwhile, the complement signaling pathway was involved in the regulation of epithelial cells on macrophages in tumor tissue. However, the CXCL signaling pathway played an important role in metastatic LN. Together, although epithelial cells in metastatic LN exhibited strong connections with immune cells and stromal cells, epithelial cells in tumors exhibited strong connections with immune checkpoint genes, which were important for tumor immunotherapy.

## Discussion

**Figure 7:**
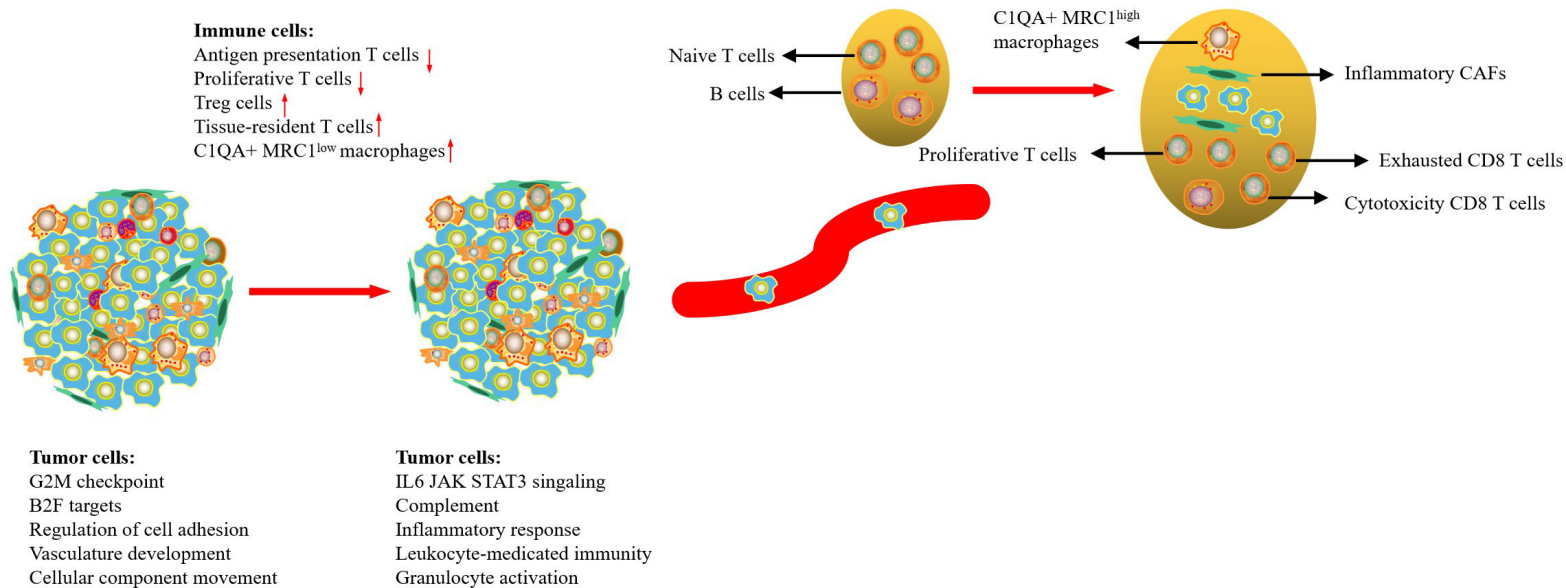
Summary of conclusion.

With the improvement of diagnostic technology and medical treatment, the prognosis of CC patients has improved significantly; however, the prognosis of patients with regional or distant metastases is still poor. In this study, we used scRNA-seq to comprehensively delineate the transcriptome characteristics of primary tumors and metastatic LN in CC. Our results indicated that tumor cells from the primary tumor and metastasis LN exhibited high CNV levels in different regions, but tumor cells from metastatic LNs were associated with high enrichment of cell cycle and immune regulation-related signaling pathways. Metastatic LN exhibited an early activated TME, characterized by an increase of proliferative T cells, NK cells, and Treg cells; exhausted CD8 T cells with cytotoxicity; and a decrease of naive T cells. We then found that metastatic LN exhibited higher expression of MRC1 than that in the tumor, so it was determined that high C1QA+MRC1^high^ macrophages might be related to tumor metastasis. Finally, we demonstrated that CAFs in metastatic LN had prominent antigen-presentation, cell adhesion, and immune regulatory functions (**Figure 7**). Our results deepened our current understanding of CC metastasis and provided novel therapeutic modalities for CC in the future.

In this study, we assessed the characteristics of tumor cells in both metastatic LNs and primary tumors. We found that tumor cells in metastatic LNs exhibited high expression of cell cycle and immune regulation-related signaling pathways. This is easy to understand because metastatic tumor cells act as endogenous antigens, effectively activating an immune response. Meanwhile, tumor cells in metastatic LNs also had high expression levels of CD47, and HLA-A/B/C, which is beneficial to tumor cells escaping the attack of immune cells. We then found that the metabolic level of tumor cells in primary lesions exhibited higher than that in metastatic LNs. Surprisingly, only bile acid biosynthesis was high in metastasis LNs. Lee et al. reported that LN metastasis requires tumor cells to undergo a metabolic shift to fatty acid oxidation (FAO), mediated by a selectively activated transcriptional co-activator Yes-associated protein (YAP) (33). The conclusion was also confirmed by Li et al., who reported immune regulation and metabolic switch in tumor-draining lymph nodes (34). Another key finding was the recognition of EMT programs in metastatic tumor cells. This program involved the upregulation of certain mesenchymal genes (CDH3, ECM1, TGFB1, WARS, and VIM). EMT is fundamental to tumor development, wound healing, and tumor cell metastasis (35). During EMT, epithelial cells lose their typical polarized states and transition to a more mobile mesenchymal phenotype. It has been hypothesized that a dynamic EMT state confers invasive properties without losing tumor-initiation capacity (36).

Many studies have demonstrated that most solid tumors have immunosuppressive TME, which is related to tumor progression (37). Similar to these findings, our previous study also showed the presence of immunosuppressive TME in CC that markedly infiltrated exhausted CD8 T cells. In this study, we confirmed that metastatic LNs exhibited an early activated state of the immune response, characterized by an increase of proliferative T cells, NK cells, and exhausted CD8 T cells with cytotoxicity, and a decrease of naive T cells compared with normal LNs (38). Another important characteristic of metastatic LN TME was the high infiltration of FOXP3+ Tregs. Treg cells usually play an immunosuppressive role in TME, attenuating an antitumor -specific immune response and thereby promoting the immune escape of tumor cells (39). Thus, in metastatic LNs, there is a balance between cytotoxicity and immunosuppressive cells. Tumor cells can not only promote the migration and proliferation of cytotoxic T cells but also induce immune tolerance of immune cells, allowing tumor cells to evade immune surveillance; surviving tumor cells further create a new microenvironment suitable for tumor growth (40).

Tumor-associated macrophages (TAMs) play a critical role in tumor growth, angiogenesis, invasion, and metastasis (41). Recently, an increasing number of scRNA-seq studies have indicated that macrophages cannot be simply divided into M1 and M2 macrophages (42, 43). Our previous study reported that macrophages from a tumor or metastatic LNs can be defined as C1QA+ macrophages, which had a dominant M2-like phenotype and immunosuppressive function (12). However, we cannot distinguish their differences in primary tumor and metastatic LNs. In this study, we found that the expression level of MRC1 (CD206) in metastatic LNs was higher than that in tumors. Therefore, the type of C1QA+ macrophages in metastatic LNs can be defined as C1QA+ MRC1^high^ macrophages. Functional analysis showed that this type of macrophage had high enrichment of phagocytosis, complement activation, and membrane invagination, indicating that immunosuppressive macrophages could be reactivated or attracted to the lesions.

Increasing evidence suggests that CAFs play an important role in cancer progression (16). CAFs are a key component of TME and have specific functions, including matrix deposition and remodeling, extensive reciprocal signaling interactions with cancer cells, and crosstalk with immunity (44). In this study, we found that tumor-derived CAFs were associated with multiple signaling pathways, such as hypoxia (HIF1A), TGF-β (TGF1 and VEGFA), and WNT/SMAD signaling pathways (WNT5A, and SMAD1) and exhibited similar functions to tumor cells, indicating that CAFs in tumors play an important role in tumor development. However, metastatic LN-derived CAFs had immunomodulatory effects with high expression levels of CD74, C7, CXCL12, S100A7, and GNLY. It is reported that CAFs can secrete various cytokines (IL-10, TGF-β, TNF, IFN-γ, and IL-6) and are involved in the recruitment and maturation of macrophages, T lymphocytes, and natural killer cells, which collectively promote aggressive behavior, attenuate the response to therapy, and increase tumor cell survival (45–47). However, some studies have confirmed that immune activation of CAFs favors the availability of the tumor to immune cells and promotes antitumor immune response (48, 49). The inhibitory or promoting effects of CAFs only occurred in specific differentiated cancers or at different stages of tumor progression. Further investigation of these features of protumorigenic and antitumorigenic functions of CAFs may bring new perspectives for future targeted therapies.

Immune checkpoint blockade (ICB) therapy, which involves the use of antibodies that target different factors of immune checkpoint pathways, has emerged as a remarkable and potential strategy for cancer treatment (50). For advanced CC, ICB therapy is undoubtedly an important and promising strategy. However, the various efficacies among patients suggest the need to identify which patients are likely to benefit from ICBs. Expression of immune checkpoint genes, such as PDCD1 (PD-1), HAVCR2, and CTLA4 are commonly used to identify suitable patients. However, we found that the expression levels of PDCD1, CTLA4, LAG3, TIGIT, BTLA, and CD40 were low in most CC primary tumors or metastatic LNs. Therefore, it is far from enough to evaluate immunotherapy solely on the expression of immune checkpoint genes. Future studies are needed to explore new markers to evaluate the effect of immunotherapy. In recent years, much progress has been made in understanding the role of natural killer (NK) cells in the tumor and viral immunity (51). NK cells are cytotoxic innate lymphoid cells that produce inflammatory cytokines and chemokines. By lysing transformed or infected cells, they limit tumor growth and viral infections (52). Given the specific TME of metastatic LNs, NK cell based on immune therapy provided a complementary therapeutic modality for CC metastasis. However, our study also had some limitations, such as relatively low coverage of 3’ end sequencing and a limited sample size. Therefore, these results should be interpreted with caution. Nevertheless, unlike published studies, this study provides, to our knowledge, an important understanding of tumors and the immune and mesenchymal cells of metastatic CC.

In summary, our data provided a valuable resource for deciphering the comprehensive gene expression landscape of tumor and TME of primary and metastatic lesions in CC. Our study provided new insights into CC metastasis biology and will promote individualized treatment for patients with CC metastasis.

## Data availability statement

The data presented in the study are deposited in the in the ArrayExpress database with accession of E-MTAB-12305. Any other data are available from the corresponding author on reasonable request. Software and resources used for analysis and plotting are described in each method section.

## Ethics statement

This study was approved by the institutional ethics review board of Shanghai OB/GYN Hospital (Number: 2021JS-017). Written informed consent was signed for all experiments involving patients.

## Author contributions

CL, DL, and SY collected the data and did the statistical analysis. CL and KH organized and submitted the manuscript. KH guided the whole process. All authors contributed to the article and approved the submitted version.

## Supplementary material

Supplementary Figure 1 Characteristic of 22 subclusters and tSNE of cell types in five samples, with each cluster (C0-C21) **(A)**. Dot plot of representative genes in 22 clusters **(A)**. Differences in pathway activity (scored per cell by GSVA) in 22 clusters **(B)**. tSNE of all cell types in five samples, with each cell color-coded for cell types **(C)**.

Supplementary Figure 2 GO analysis of up-regulated genes in seven epithelial cell clusters.

Supplementary Figure 3 Functional analysis of up-regulated in different samples. GO analysis of up-regulated genes of tumor cells in non-metastatic tumors (tumors 1 and 2) **(A)**. GO analysis of up-regulated genes of tumor cells in metastatic tumors (tumor 3) **(B)**. Venn diagram showing the 120 overlappings of up-regulated genes in P-LN **(C)**. GO analysis of 120 up-regulated genes in P-LN **(D)**. GO analysis of up-regulated genes of tumor cells in P-LN **(E)**. GO analysis of up-regulated genes of tumor cells in tumor 3 **(F)**.

Supplementary Figure 4 Dot plot of metabolic differences of malignant cells among samples.

Supplementary Figure 5 Survival analysis of mesenchymal and epithelial markers according to TCGA data.

Supplementary Figure 6 Comparison of cell number, gene expression, and functional status of NK/T cells between metastatic tumor (tumor 3) and non-metastatic tumors (tumors 1 and 2). The difference in cell number of NK/T cells between metastatic tumor (tumor 3) and non-metastatic tumors (tumors 1 and 2) **(A)**. Volcano plot showing differentially expressed genes of NK/T cells between metastatic tumor (tumor 3) and non-metastatic tumors (tumors 1 and 2) **(B)**. GO analysis of upregulated genes of NK/T cells in non-metastatic tumor **(C)**. GO analysis of upregulated genes of NK/T cells in metastatic tumor **(D)**.

Supplementary Figure 7 GO analysis of up-regulated genes in nine macrophage clusters.

Supplementary Figure 8 Comparison of functional status of macrophages among different groups. GO analysis of up-regulated genes of macrophage in non metastatic tumor **(A)**. GO analysis of up-regulated genes of macrophage in metastatic tumor **(B)**. Comparison of M2- and M1-signature score between metastatic tumor (tumor 3) and non-metastatic tumors (tumor 1 and 2) **(C)**. GO analysis of up-regulated genes of macrophage in P-LN **(D)**. GO analysis of up-regulated genes of macrophage in N-LN **(E)**. The comparison of M2- and M1-signature score between P-LN and N-LN **(F)**.

Supplementary Figure 9 GO analysis of up-regulated genes in six CAF clusters.

Supplementary Figure 10 Comparison of functional status of CAFs among different groups. GO analysis of up-regulated genes of CAFs in non-metastatic tumor **(A)**. GO analysis of up-regulated genes of CAFs in metastatic tumor **(B)**. GO analysis of up-regulated genes of CAFs in P-LN **(C)**. GO analysis of up-regulated genes of CAFs in N-LN **(D)**.
