# Supplementary material for: Integrated single-cell transcriptome analysis of the tumor ecosystems underlying cervical cancer metastasis
Source: Front Immunol. 2022 Dec 9;13:966291. doi: 10.3389/fimmu.2022.966291 (PMC9780385; doi:10.3389/fimmu.2022.966291)
Supplement: Supplementary file 3 [file DataSheet_1.doc]

library(Seurat)

pbmc <- Read10X(data.dir = dir)

pbmc <- CreateSeuratObject(counts = pbmc,project = "seurat", min.cells = 3, min.features = 200, names.delim = "_",)

pbmc[["percent.mt"]] <- PercentageFeatureSet(object = pbmc, pattern = "^MT-")

pdf(file="04.featureViolin.pdf",width=10,height=6)

VlnPlot(object = pbmc, features = c("nFeature_RNA", "nCount_RNA", "percent.mt"), ncol = 3)

dev.off()

pbmc <- subset(x = pbmc, subset = nFeature_RNA > 50 & percent.mt < 30) pdf(file="04.featureCor.pdf",width=10,height=6)

plot1 <- FeatureScatter(object = pbmc, feature1 = "nCount_RNA", feature2 = "percent.mt",pt.size=1.5)

plot2 <- FeatureScatter(object = pbmc, feature1 = "nCount_RNA", feature2 = "nFeature_RNA",,pt.size=1.5)

CombinePlots(plots = list(plot1, plot2))

dev.off()

pbmc <- NormalizeData(object = pbmc, normalization.method = "LogNormalize", scale.factor = 10000)

pbmc <- FindVariableFeatures(object = pbmc, selection.method = "vst", nfeatures = 1500)

top10 <- head(x = VariableFeatures(object = pbmc), 10)

pdf(file="04.featureVar.pdf",width=10,height=6plot1 <- VariableFeaturePlot(object = pbmc)

plot2 <- LabelPoints(plot = plot1, points = top10, repel = TRUE)

CombinePlots(plots = list(plot1, plot2))

dev.off()

pbmc=ScaleData(pbmc)

pbmc=RunPCA(object= pbmc,npcs = 20,pc.genes=VariableFeatures(object = pbmc)) pdf(file="05.pcaGene.pdf",width=10,height=8)

VizDimLoadings(object = pbmc, dims = 1:4, reduction = "pca",nfeatures = 20)

dev.off()

pdf(file="05.PCA.pdf",width=6.5,height=6)

DimPlot(object = pbmc, reduction = "pca")

dev.off()

pdf(file="05.pcaHeatmap.pdf",width=10,height=8)

DimHeatmap(object = pbmc, dims = 1:4, cells = 500, balanced = TRUE,nfeatures = 30,ncol=2)

dev.off()

pbmc <- JackStraw(object = pbmc, num.replicate = 100)

pbmc <- ScoreJackStraw(object = pbmc, dims = 1:20)

pdf(file="05.pcaJackStraw.pdf",width=8,height=6)

JackStrawPlot(object = pbmc, dims = 1:20)

dev.off()

library(harmony)

pbmc <- RunHarmony(pbmc, "orig.ident")

names(pbmc@reductions)

pbmc <- FindNeighbors(pbmc, reduction = "harmony",dims = 1:20)

pbmc <- FindClusters(pbmc, resolution = 0.20)

pbmc <- RunTSNE(pbmc, dims = 1:20,

reduction = "harmony")

DimPlot(pbmc, reduction = 'tsne', group.by = 'orig.ident',

label = TRUE, pt.size = 0.5) + NoLegend()
