# Supplementary figures and images for: Integrated single-cell transcriptome analysis of the tumor ecosystems underlying cervical cancer metastasis
Source: Front Immunol. 2022 Dec 9;13:966291. doi: 10.3389/fimmu.2022.966291 (PMC9780385; doi:10.3389/fimmu.2022.966291)

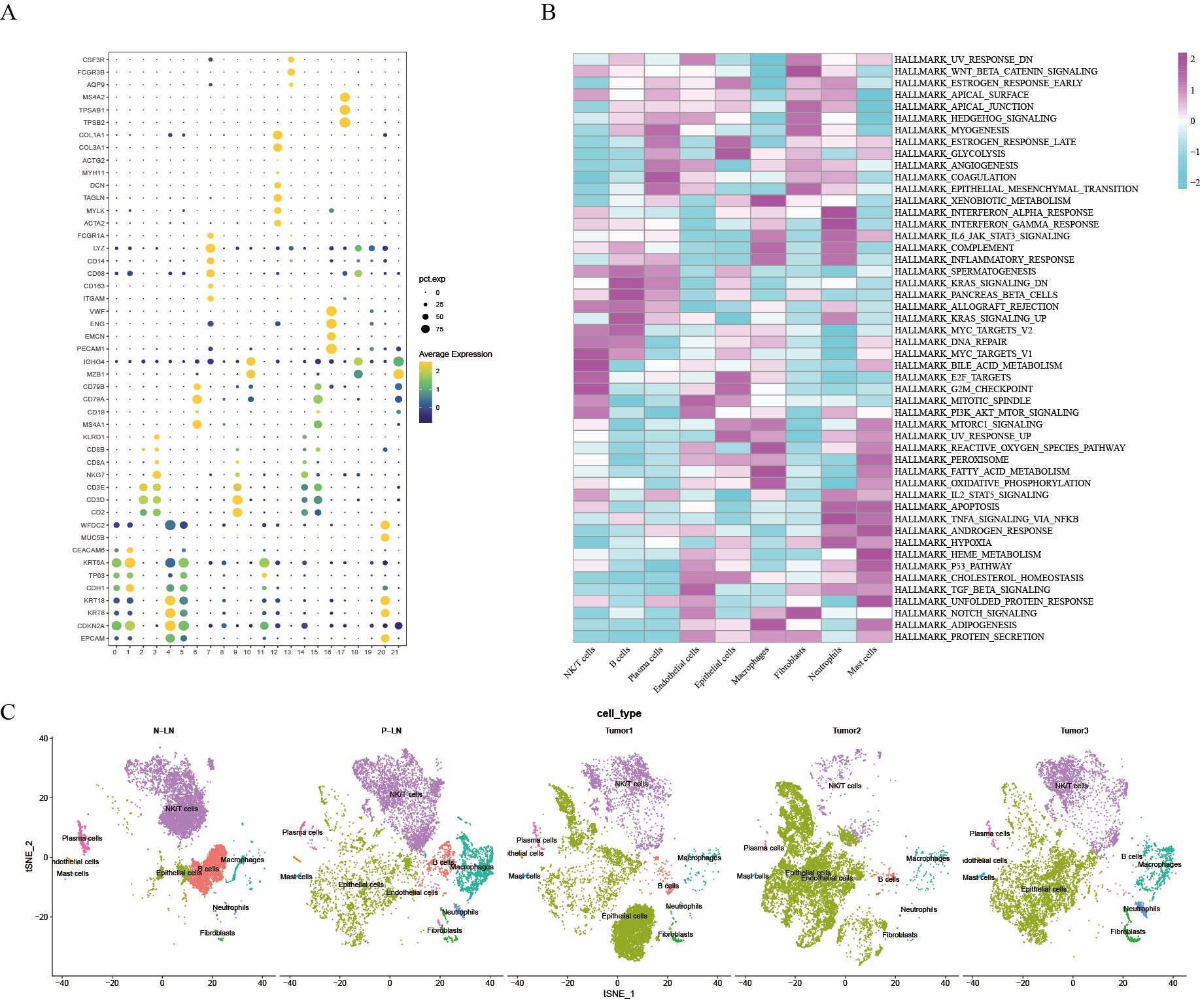

Supplement: Supplementary Figure 1 — Characteristic of 22 subclusters and tSNE of cell types in five samples, with each cluster (C0-C21) (A). Dot plot of representative genes in 22 clusters (A). Differences in pathway activity (scored per cell by GSVA) in 22 clusters (B). tSNE of all cell types in five samples, with each cell color-coded for cell types (C). [file Image_1.jpeg]

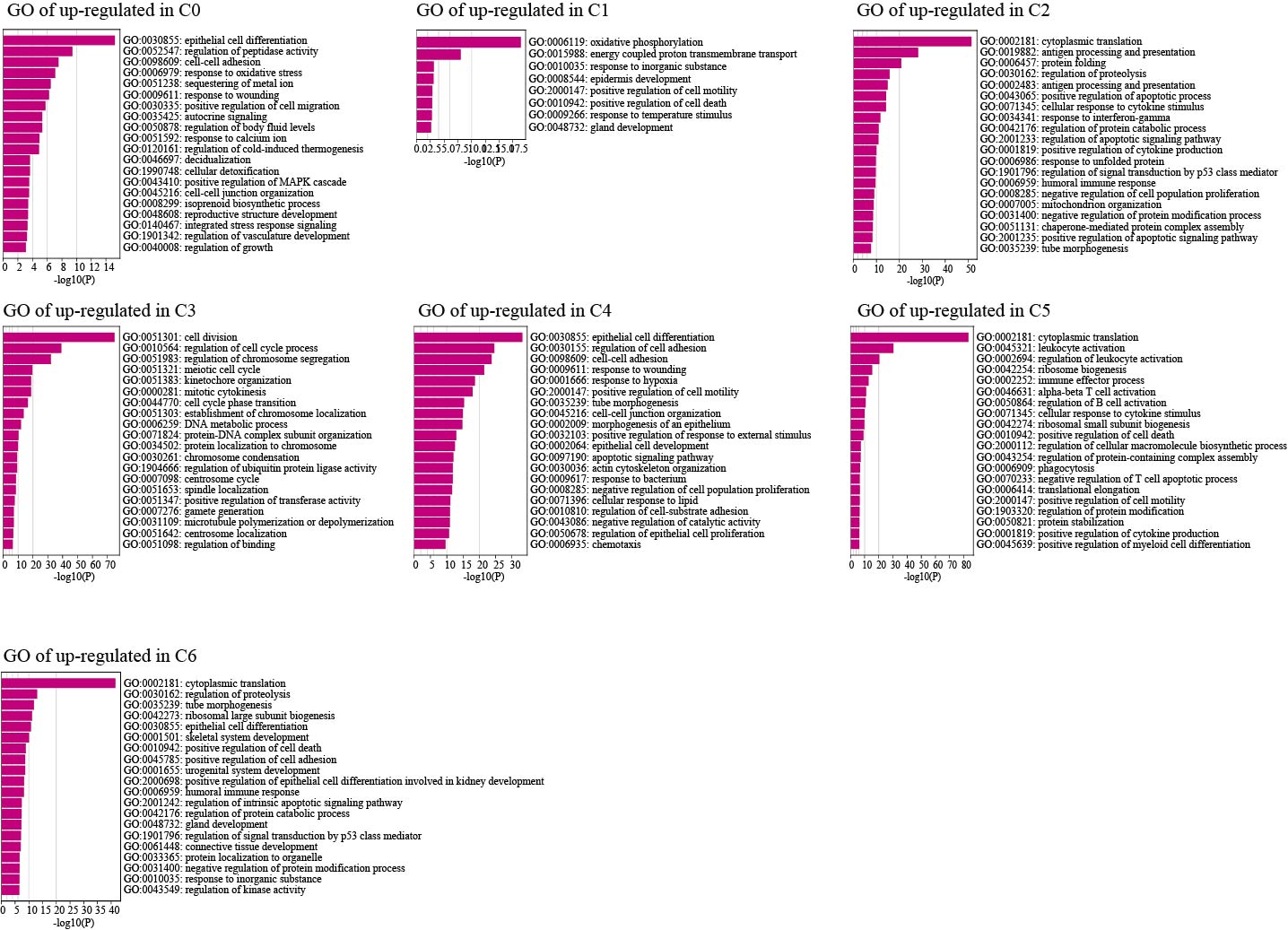

Supplement: Supplementary Figure 2 — GO analysis of up-regulated genes in seven epithelial cell clusters. [file Image_2.jpeg]

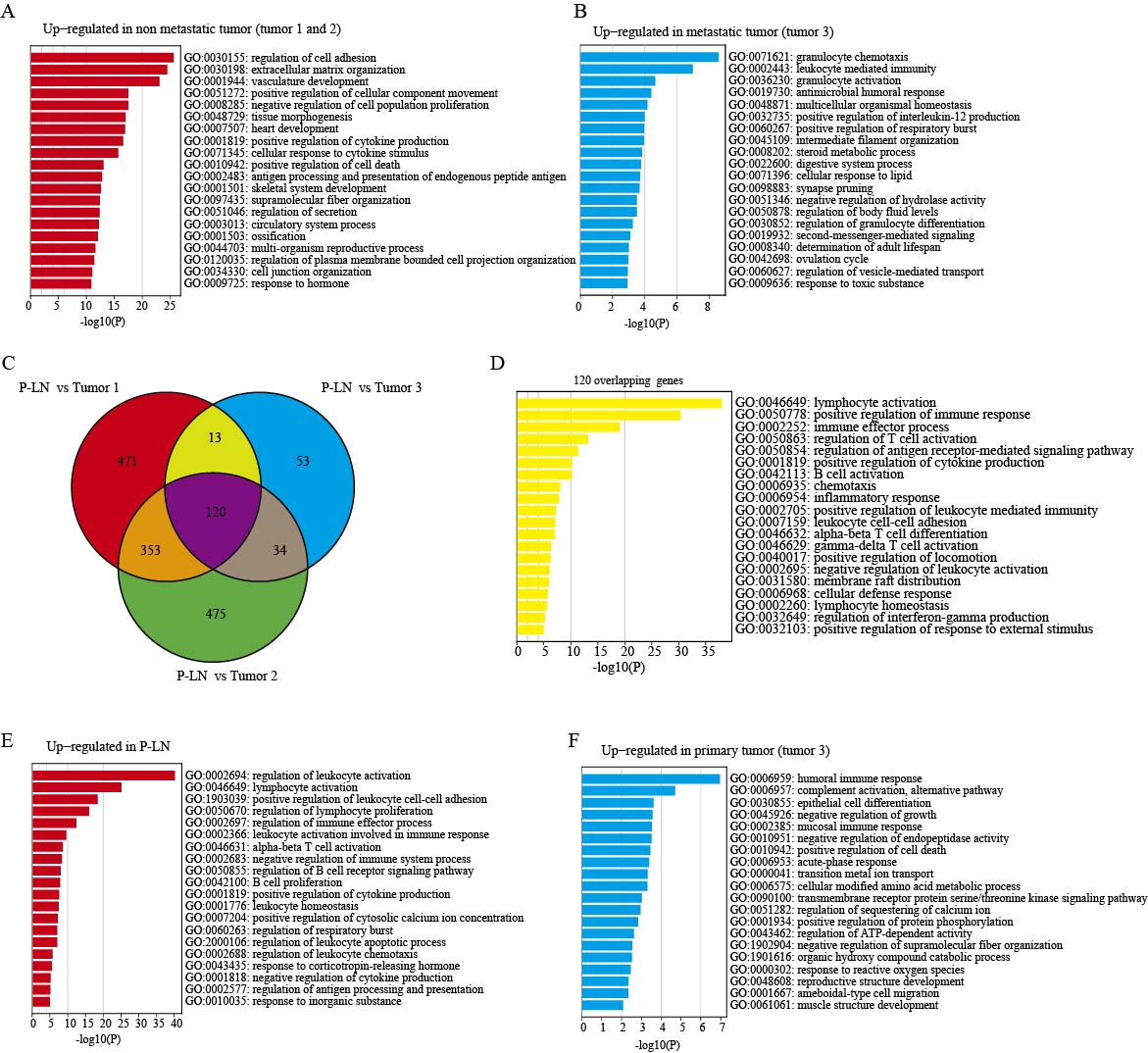

Supplement: Supplementary Figure 3 — Functional analysis of up-regulated in different samples. GO analysis of up-regulated genes of tumor cells in non-metastatic tumors (tumors 1 and 2) (A). GO analysis of up-regulated genes of tumor cells in metastatic tumors (tumor 3) (B). Venn diagram showing the 120 overlappings of up-regulated genes in P-LN (C). GO analysis of 120 up-regulated genes in P-LN (D). GO analysis of up-regulated genes of tumor cells in P-LN (E). GO analysis of up-regulated genes of tumor cells in tumor 3 (F). [file Image_3.jpeg]

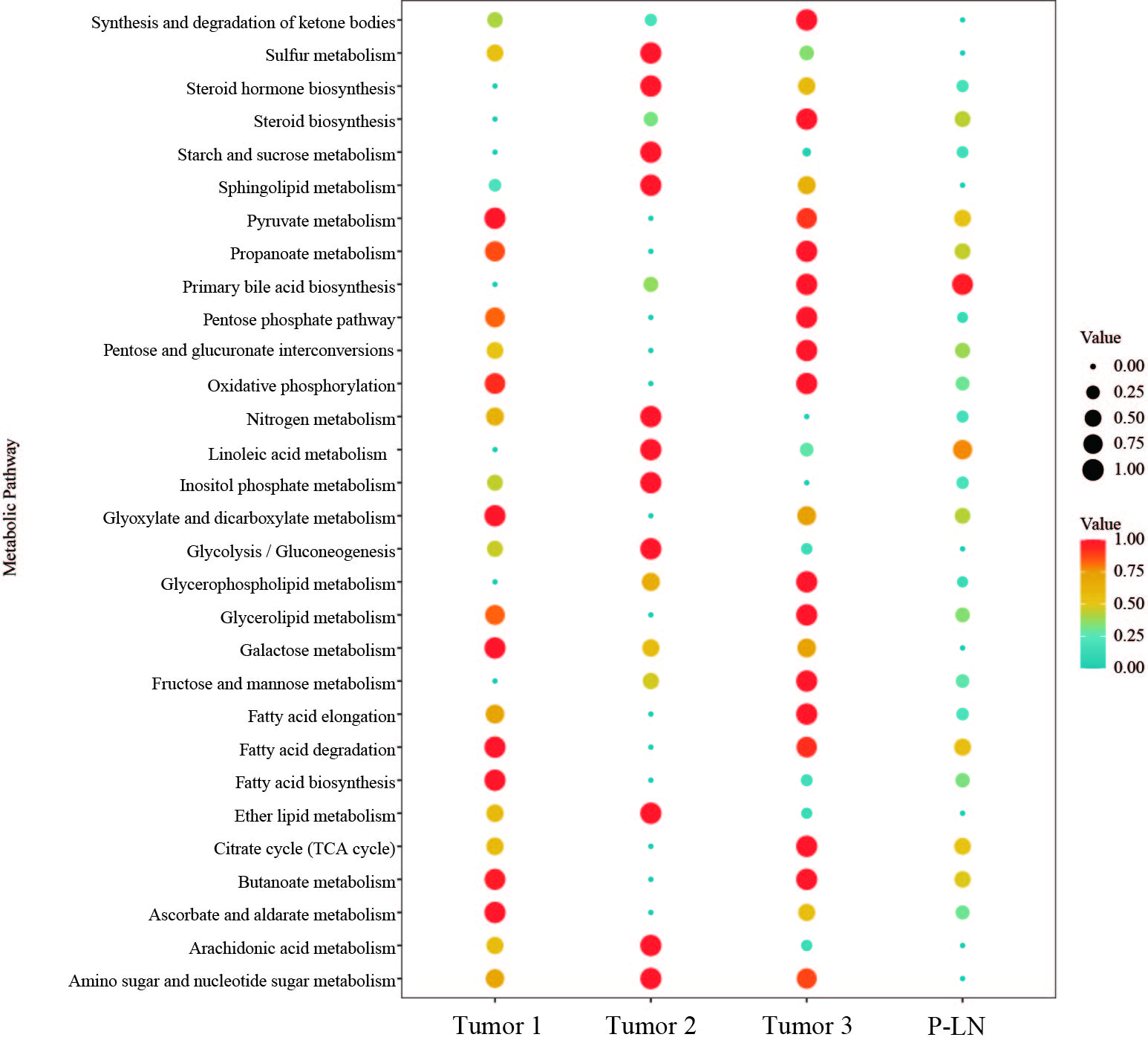

Supplement: Supplementary Figure 4 — Dot plot of metabolic differences of malignant cells among samples. [file Image_4.jpeg]

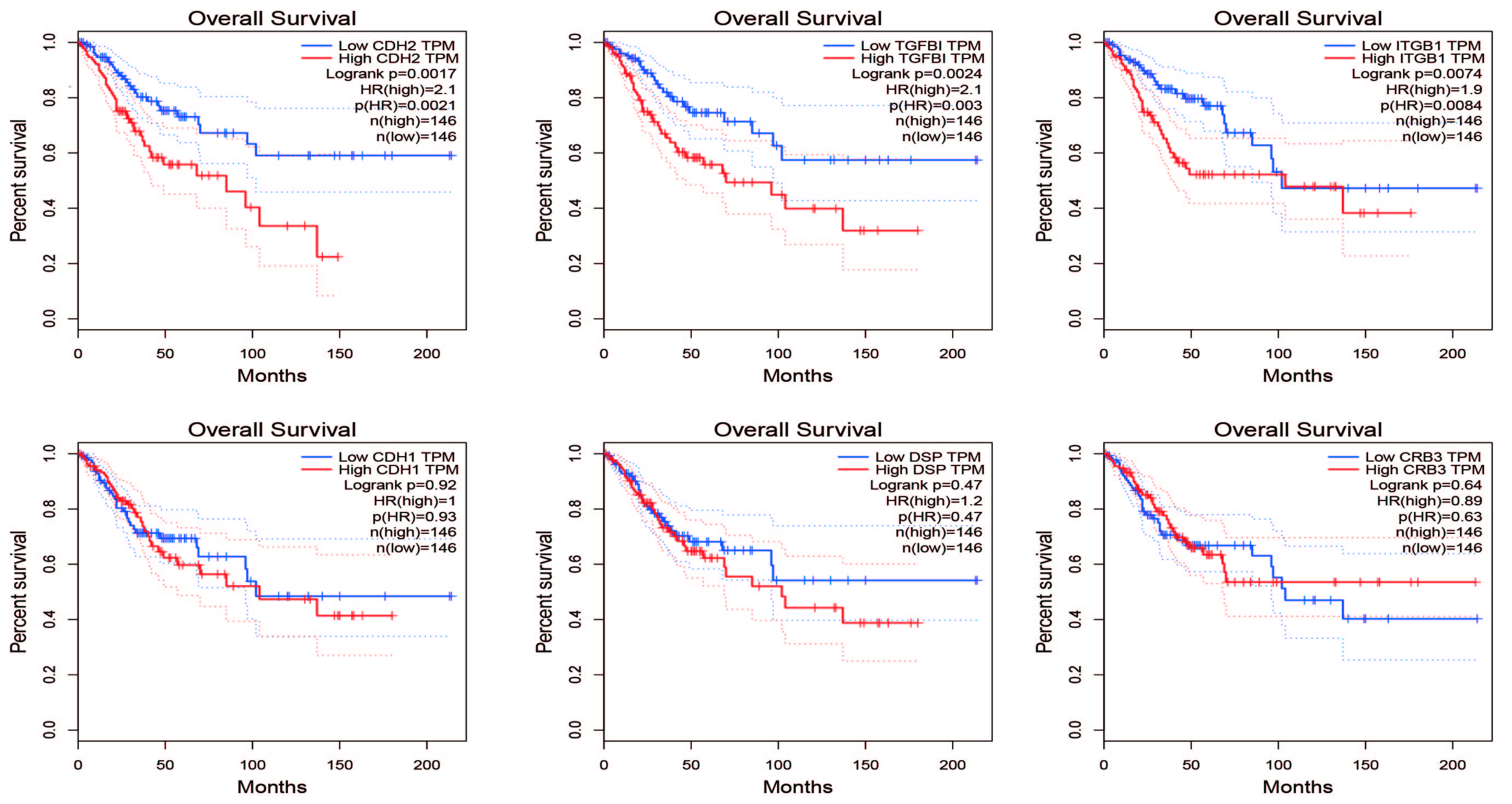

Supplement: Supplementary Figure 5 — Survival analysis of mesenchymal and epithelial markers according to TCGA data. [file Image_5.jpeg]

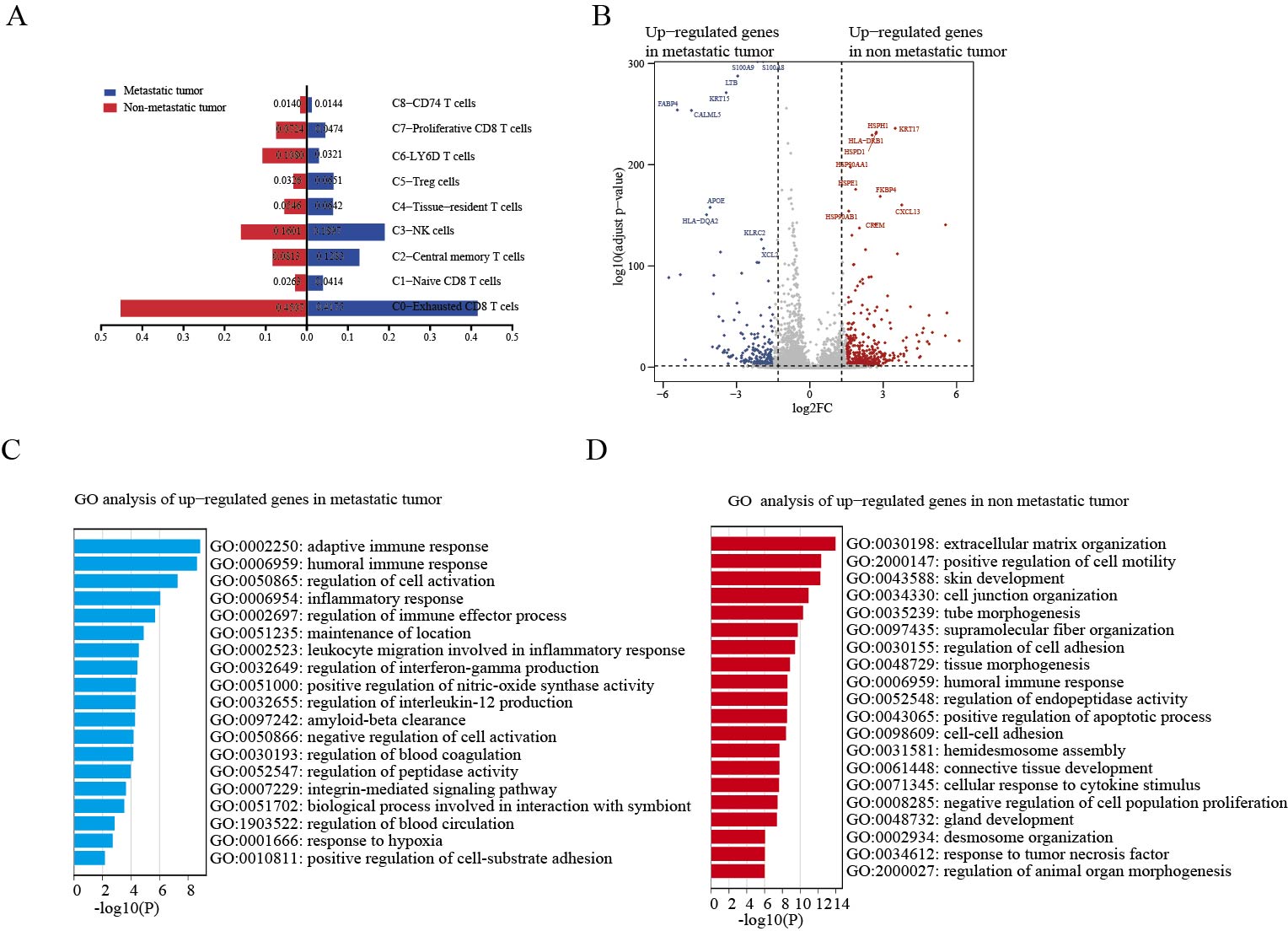

Supplement: Supplementary Figure 6 — Comparison of cell number, gene expression, and functional status of NK/T cells between metastatic tumor (tumor 3) and non-metastatic tumors (tumors 1 and 2). The difference in cell number of NK/T cells between metastatic tumor (tumor 3) and non-metastatic tumors (tumors 1 and 2) (A). Volcano plot showing differentially expressed genes of NK/T cells between metastatic tumor (tumor 3) and non-metastatic tumors (tumors 1 and 2) (B). GO analysis of upregulated genes of NK/T cells in non-metastatic tumor (C). GO analysis of upregulated genes of NK/T cells in metastatic tumor (D). [file Image_6.jpeg]

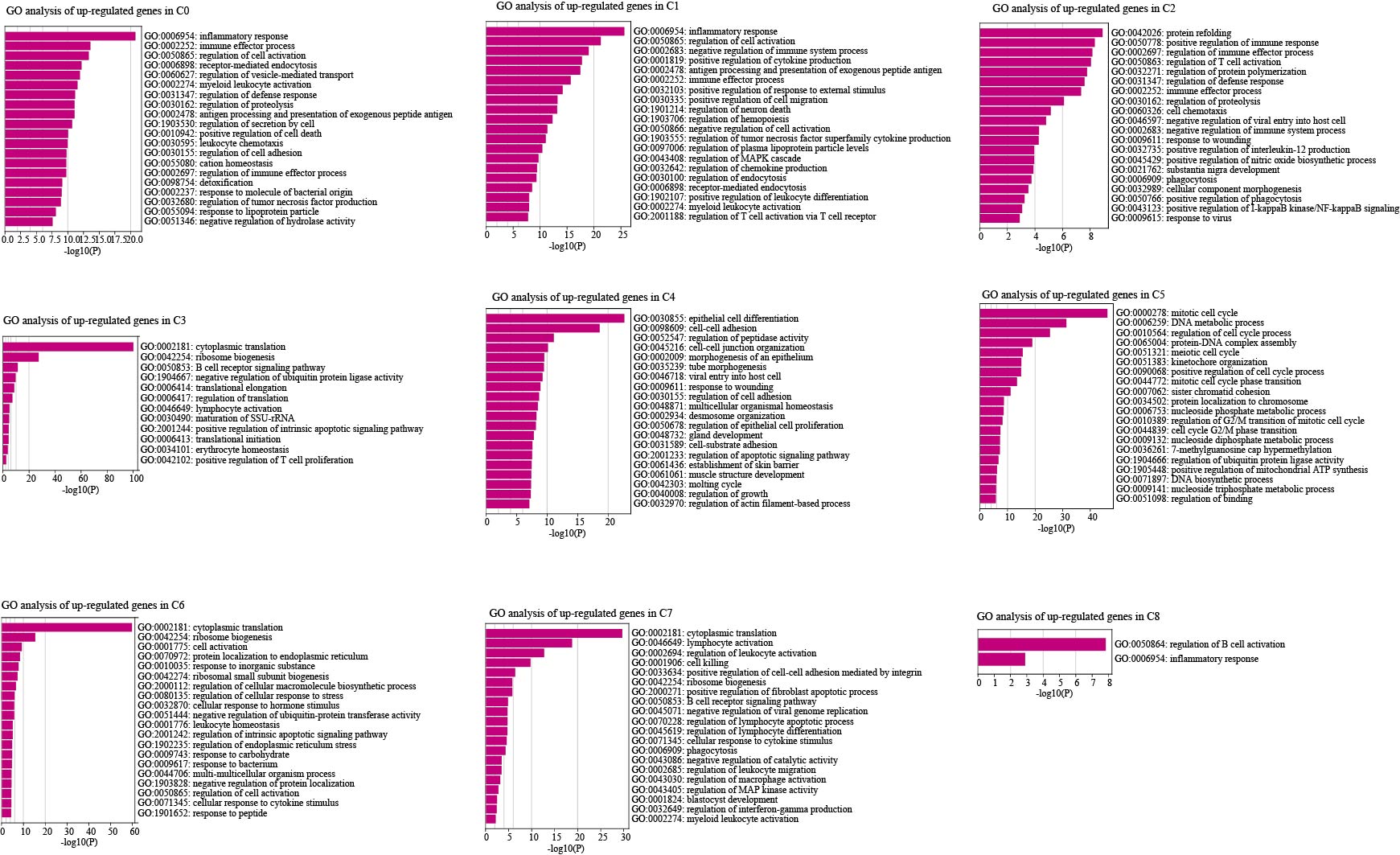

Supplement: Supplementary Figure 7 — GO analysis of up-regulated genes in nine macrophage clusters. [file Image_7.jpeg]

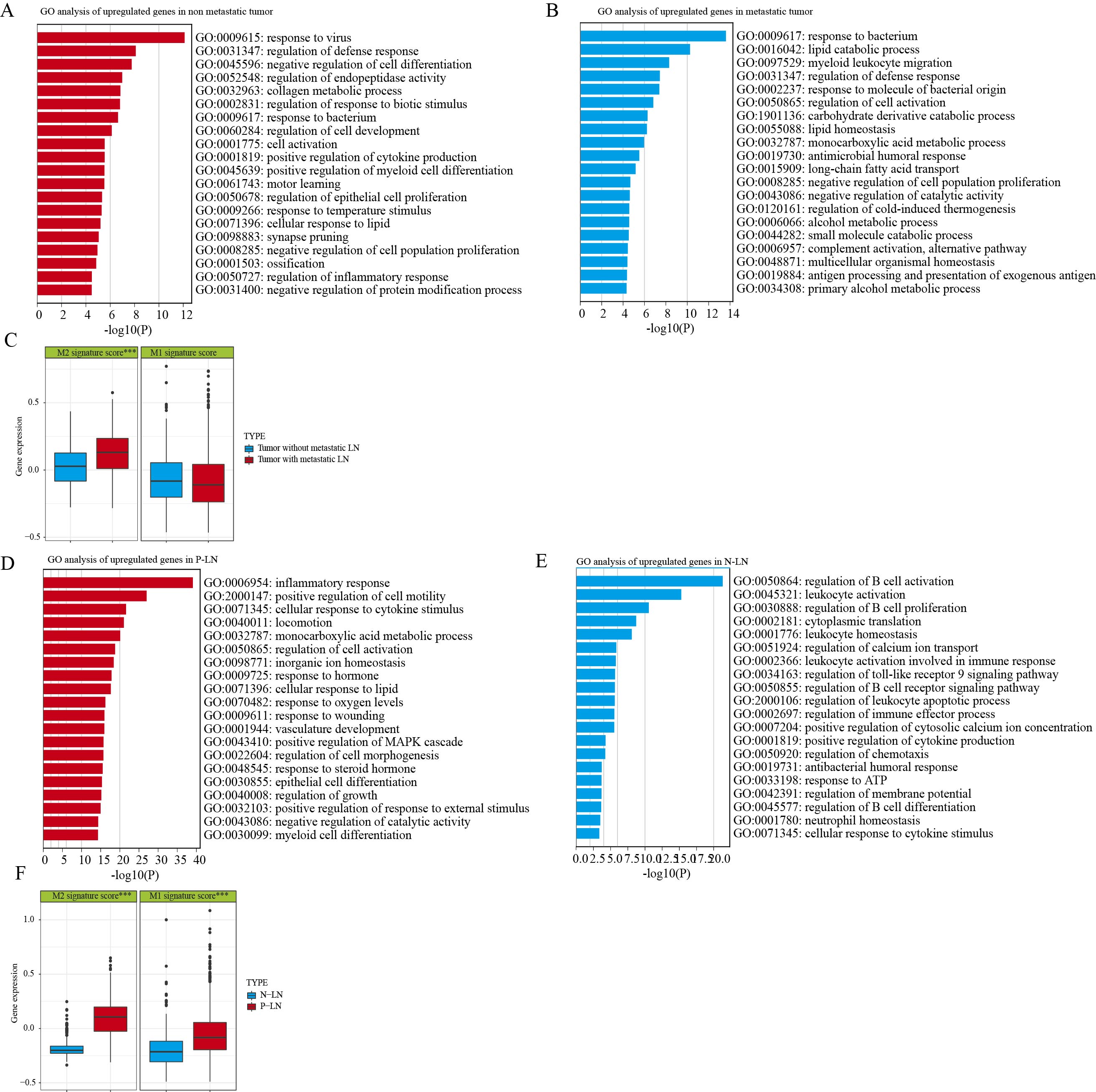

Supplement: Supplementary Figure 8 — Comparison of functional status of macrophages among different groups. GO analysis of up-regulated genes of macrophage in non metastatic tumor (A). GO analysis of up-regulated genes of macrophage in metastatic tumor (B). Comparison of M2- and M1-signature score between metastatic tumor (tumor 3) and non-metastatic tumors (tumor 1 and 2) (C). GO analysis of up-regulated genes of macrophage in P-LN (D). GO analysis of up-regulated genes of macrophage in N-LN (E). The comparison of M2- and M1-signature score between P-LN and N-LN (F). [file Image_8.jpeg]

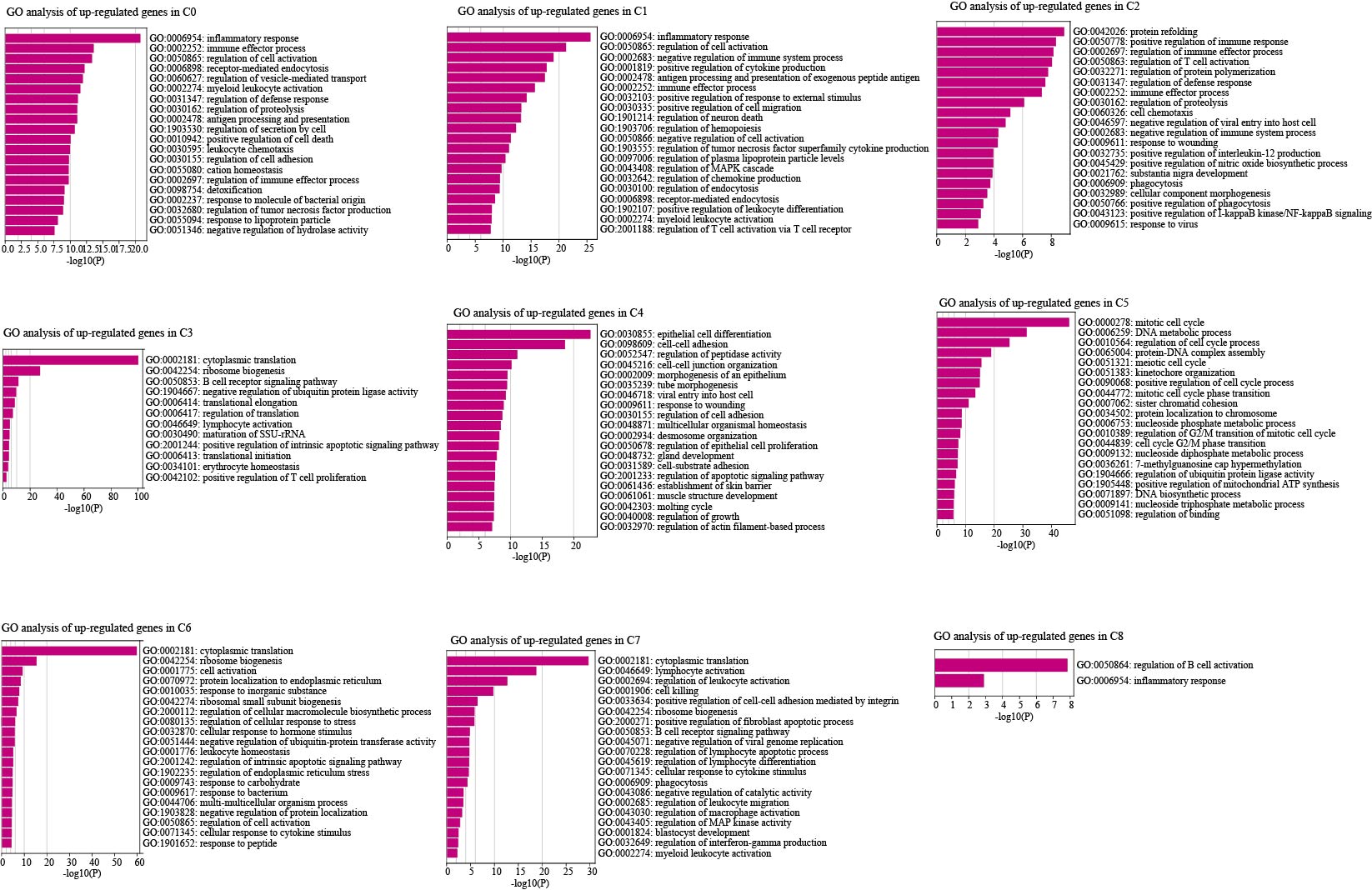

Supplement: Supplementary Figure 9 — GO analysis of up-regulated genes in six CAF clusters. [file Image_9.jpeg]

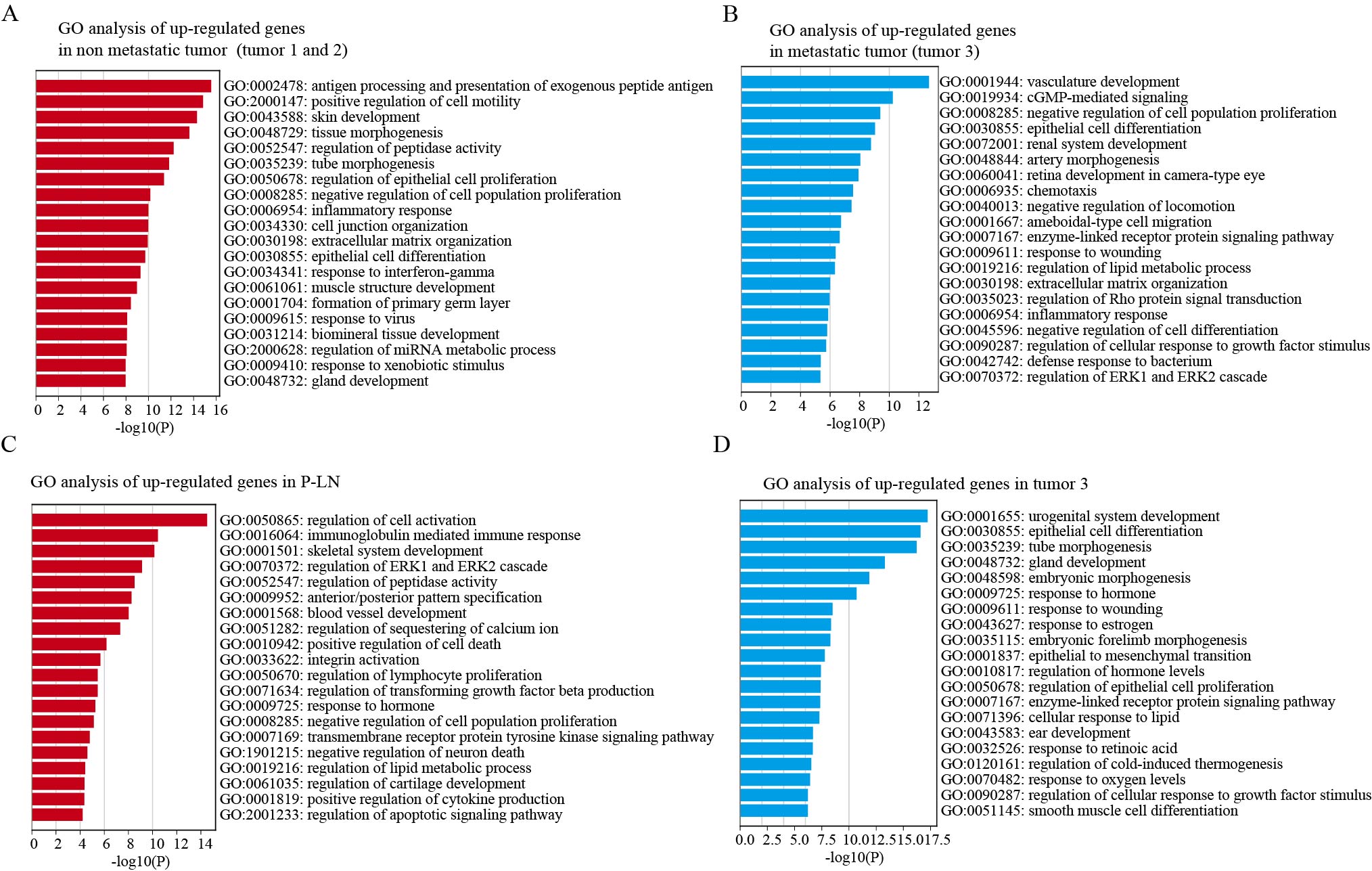

Supplement: Supplementary Figure 10 — Comparison of functional status of CAFs among different groups. GO analysis of up-regulated genes of CAFs in non-metastatic tumor (A). GO analysis of up-regulated genes of CAFs in metastatic tumor (B). GO analysis of up-regulated genes of CAFs in P-LN (C). GO analysis of up-regulated genes of CAFs in N-LN (D). [file Image_10.jpeg]
